# Engineering Catalytic Efficiency by Thiolate-Protected Trimetallic (Cu, Pd, Au) Nanoclusters: Single-Atom Alloy Catalysts for Water–Gas Shift

**DOI:** 10.1021/acscatal.5c04165

**Published:** 2025-08-22

**Authors:** Stephan Pollitt, Thomas Haunold, Sakiat Hossain, Gereon Behrendt, Michael Stöger-Pollach, Tokuhisa Kawawaki, Noelia Barrabés, Malte Behrens, Yuichi Negishi, Günther Rupprechter

**Affiliations:** † Institute of Materials Chemistry, 27259 TU Wien, Getreidemarkt 9/BC, 1060 Vienna, Austria; ‡ Department of Applied Chemistry, Faculty of Science, 13101 Tokyo University of Science, Kagurazaka, Shinjuku-ku, Tokyo 162-8601, Japan; § Institute of Inorganic Chemistry, Solid State Chemistry and Catalysis, Kiel University, Max-Eyth-Str. 2, 24118 Kiel, Germany; ∥ University Service Center for Transmission Electron Microscopy (USTEM), 27259 TU Wien, Stadionallee 2/057-02, 1020 Vienna, Austria

**Keywords:** single-atom alloy catalysts, water−gas shift reaction, thiolate-protected nanoclusters, trimetallic nanoclusters, SMSI

## Abstract

The “crude oil exodus” and energy transition will finally hinge on the availability of hydrogen. Catalytic processes like the water–gas shift (WGS) reaction may significantly contribute to its production and become crucial for utilizing alternative feedstocks. This work demonstrates how thiolate-protected gold nanoclusters can be employed as precursors for single-atom alloy (SAA) catalysts. The clusters serve as carriers of heteroatom dopants (Cu, Pd) while precisely maintaining 25 metal atoms per cluster (<1 nm). Using the 2PET ligand during synthesis led to high yield and cluster stability, but ligand exchange was required to link clusters to a ZnO support efficiently. Introducing pMBA as a ligand enabled a homogeneous cluster distribution on the ZnO surface, creating a well-defined catalyst with dual functionality. This SAA catalyst, outperforming a Cu/ZnO/Al_2_O_3_ benchmark in WGS, may get industrial relevance when upscaled while still serving as a well-defined model system in catalysis. Thereby, it bridges the gap between practical applications and fundamental research. Pre- and postreaction analysis by XPS proved the presence of the dopants in the catalysts in the expected stoichiometry, showed changes in the electronic structures, but also revealed sulfur migration from the clusters/ligands to the support, forming ZnS. Furthermore, XPS unveiled a pretreatment-induced SMSI decoration effect, stabilizing the small particles during catalysis. (S)­TEM indicated a homogeneous cluster distribution on ZnO after synthesis and proved small particle sizes throughout the experiments. In situ DRIFTS confirmed the accessibility of the dopant atoms by the reactant CO and also detected adsorbed byproducts. The precise size and doping control of thiolate-protected SAA nanoclusters, together with their catalytic performance, demonstrate the potential for targeted future investigations in a wide range of industrial applications.

## Graphical abstract

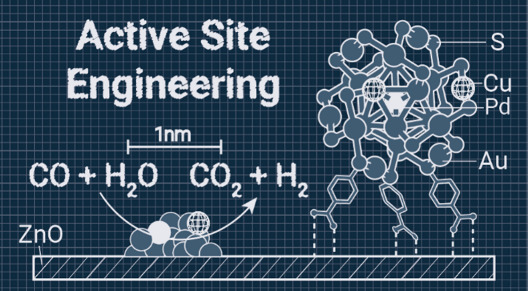

## Introduction

Upon transitioning toward eco-friendly industrial processes, pursuing alternative fuels and sustainable feedstocks for fine chemicals is a prerequisite for such a paradigm shift. Hydrogen emerges as a highly promising energy carrier, prompting extensive research into its clean production and storage as a strategic move away from reliance on crude oil.^1^ To date, the primary source of hydrogen is syngas (CO + H_2_) obtained from methane steam reforming (MSR) or methane dry reforming (MDR).^1–6^ Similar feed product mixtures are obtained when employing more sustainable feedstocks like biomass, food waste, wastewater, or methane from dairy farms (although often requiring additional purification).^7^

However, the carbon monoxide in the syngas poses challenges for using H_2_ in various chemical processes and applications, such as fuel cells or catalytic reactions, where it functions as a detrimental poison and necessitates prior removal.^8–11^ This can be achieved via the water–gas shift reaction (e.g., carried out following MSR or MDR), wherein carbon monoxide reacts with water to form carbon dioxide and hydrogen, reducing the CO concentration and increasing the hydrogen content_._ ^12–14^ Noteworthy, an efficient catalyst is required. The industrial benchmark for low-temperature WGS is copper (Cu) supported on zinc oxide, further promoted by alumina (Cu/ZnO/Al_2_O_3_).^12,15,16^ Efforts to explore more potent catalysts, such as copper supported on cerium dioxide (CeO_2_) or noble metals like gold (Au) or palladium (Pd), have demonstrated their higher activity.^14,17–19^ Nevertheless, stability-related obstacles, such as agglomeration of nanoparticles, the high cost of noble metals, and their susceptibility to coking, have impeded their widespread implementation in technological WGS. In any case, the activity of WGS catalysts seems to originate particularly from low-coordinated sites, with defect/step/corner sites being crucial.^20,21^ Thus, the quest for a WGS catalyst with exceptional activity paired with long-term stability remains a vivid research area.

A promising strategy is to use single-atom catalysts (SACs), that drastically reduce the noble metal loading while maintaining a high number of active sites.^22–27^ In single-site catalysts, the active sites consist of single metal atoms dispersed on/in an organic or inorganic host, e.g., metal oxides, carbon supports, or porphyrins.^24,28–39^

Single-atom-alloys, however, feature an isolated active element (atom) residing within a metal host matrix.^40–45^ This configuration imparts unique properties, such as alterations in the electronic structure of the active atom through electron transfer or the localized separation of intermediate states of the reactants, e.g., adsorption, dissociation, and the transition state between the host and the active element, resulting in improved activity and selectivity in hydrogenation and oxidation reactions.^46,47^ Furthermore, catalyst deactivation due to e.g., CO poisoning or coking can be prevented by the SAA approach.^48,49^ Using Monte Carlo simulations, Svensson and Grönbeck demonstrated a site communication effect of single Pd atoms in the Au host environment, steering the catalytic activity toward direct H_2_O_2_ production.^50^ Site communication was also reported for isolated La atoms on Rh.^51^ Confined initially to Ultra-High Vacuum (UHV) research, advances in synthesis strategies have facilitated the seamless integration of single-atom-alloy catalysis into nanoparticle research.^40^ Techniques such as sequential reduction, incipient wetness impregnation, strong electrostatic adsorption, controlled surface reactions, electroless deposition, and coreduction, among others, have been employed.^42,52,53^ Each technique presents distinct advantages and drawbacks concerning control, experimental complexity, and the homogeneity of the desired single-atom-alloy configuration.

Heteroatom-doped thiolate-protected gold nanoclusters are particularly promising as single-atom alloy catalysts, owing to recent breakthroughs in synthesis yields.^54–58^ They offer advantages over the synthesis methods mentioned above due to the inherent atomic precision and homogeneity.^59^ This precision is achieved by forming magic atom-numbered Au clusters (e.g., Au = 11, 25, 38, 102, 144,···) stabilized against agglomeration/decomposition by a ligand shell.^60^ Various magic numbers and ligands facilitate fine-tuning of cluster structures tailored to specific reactions. Moreover, dopant atoms can be introduced by coreduction, by substitution from a metalorganic complex or from surface atoms of metal foils, further expanding the pathways for designing targeted catalyst structures.^54,61–67^ Once clusters have been immobilized on a support material, a mild thermal pretreatment removes the ligand shell facilitating interactions of reactants with the active metal sites.^68–71^ Alternatively, exposure to light or oxidative/reductive chemical approaches can be performed to remove the ligands from the clusters.^72,73^ Due to the small cluster size (approximately 1 nm), nearly all atoms are situated on the surface, and the educts can adsorb on the heteroatom dopants unhindered. Regarding scalability, recent advances in the synthesis of ligand-protected clusters have led to significant improvements in yield, reproducibility, and ligand-exchange procedures. Although not yet standard in industrial settings, these developments indicate that the approach is becoming increasingly suitable for larger-scale or semiautomated synthesis, especially within academic and specialized research environments.^54–58^

Schumann et al. recently reported a ten-electron count rule to predict optimal dopant and adsorbate interaction for SAAs with a Cu, Ag, or Au host.^74^ In the case of the water–gas shift reaction, interaction with CO and H_2_O is required. H_2_O is known to adsorb via electrostatic interaction, whereas CO prefers chemical bonding. Based on the ten-electron count rule, 4d and 5d group VIII elements should be beneficial for WGS, e.g., by doping Au clusters with Pd, promoting interactions with CO.^71^ Additional doping with Cu, due to its established role in WGS, is expected to have a further promotional effect on the synergistic Au–Pd system.

Notably, in 2009 Fields-Zinna et al. and Negishi et al. independently reported the successful synthesis of bimetallic PdAu_24_(2PET)_18_ (2PET: −SC_2_H_4_Ph, 2-phenylethylthiolate) clusters using the Brust method, providing the basis of follow-up research.^75–77^ Subsequently, Negishi et al. reported stable bimetallic Cu_*x*_Au_25–*x*_(2PET)_18_ (*x* = 1–5) clusters, where copper significantly influenced the redox potential and optical properties of the clusters.^63^ However, the Cu dopants induced a geometrical distortion of the cluster structure, limiting the maximum number of dopants to *n* ≤ 5 and decreasing the stability against degradation in solution. Concerning trimetallic clusters, Sharma et al. reported that controlled Cu doping was most effectively achieved for a PdAu_24_(2PET)_18_ cluster due to stability reasons, allowing the introduction of up to 3 Cu atoms. In combination with the Pd center atom, higher Cu numbers strain the cluster structure, again leading to degradation.^78,79^ Of note, Pd was also identified as having a beneficial effect on cluster stability and WGS activity.^80,81^

Apart from the metal atoms or particles in heterogeneous catalysts, the support can also play a key role, for example, in stabilizing the active site.^14,18,82,83^ Recent reports of cluster catalysis emphasize stability issues, though. They describe the structural properties as dynamic, leading to agglomeration, especially at elevated temperatures.^68,84,85^ Different approaches have been used to stabilize small nanoparticles: using support materials with numerous defect sites like CeO_2_ or mixed oxides, anchoring on N-doped carbon supports, and atomic layer deposition (ALD) to partially cover particles with a metal oxide.^14,86–88^ Some reducible metal oxides, such as TiO_2_, CeO_2_, or V_2_O_3_, exhibit the classical SMSI effect with group VIII metals.^89,90^ Under reductive conditions, (sub)­oxides of the support migrate on top of the nanoparticles, forming layers alike ALD. ZnO and TiO_2_ supports were reported to induce SMSI on Au, which may be utilized for stabilization.^91–93^

Consequently, a trimetallic cluster Cu_*x*_PdAu_24–*x*_(2PET)_18_ supported on ZnO was selected for the current investigations and was compared to its mono- and bimetallic counterparts. For further comparison, a conventional Cu/ZnO/Al_2_O_3_ catalyst was included as a benchmark to contextualize the performance of the atomically precise SAA systems studied here, with the focus placed on understanding structure–activity relationships at the atomic scale.^15,21^ Following the strategy discussed above, we synthesized mono-, bi-, and trimetallic thiolate-protected gold nanoclusters as precursors for SAAs in the form of Au (host), Cu, or/and Pd dopants. The clusters were subsequently supported on a highly stabilizing support to maintain the SAAs in the low nm size range, as characterized by scanning transmission electron microscopy ((S)­TEM) and X-ray photoelectron spectroscopy (XPS).^94^ SAAs were activated by mild oxidative and subsequent reductive thermal treatments. The presence and accessibility of single sites were demonstrated by CO adsorption monitored by in situ DRIFTS. The SAAs were tested in the water–gas shift reaction, in which they outperformed a technical benchmark catalyst. Furthermore, post-pretreatment and post-reaction characterization revealed at least partial encapsulation via an SMSI effect, which stabilized the tiny clusters, and XPS demonstrated changes in the chemical states that affected reaction behavior.

## Results and Discussion

### Precursor Synthesis for Single-Atom Alloy Catalysts and Ligand Exchange for Improved Immobilization

Thiolate-protected gold nanoclusters Au_25_(2PET)_18_ and PdAu_24_(2PET)_18_ were synthesized according to previously established procedures, described in the Supporting Information (SI). Purity and synthesis success were confirmed by MALDI-MS and UV/vis absorption spectroscopy in Figures S1–S3.^61,77^ These clusters served as basic building blocks for Cu doping, yielding bimetallic Cu_*x*_Au_25–*x*_(2PET)_18_ (*x* = 1, 2, 3) and the desired trimetallic Cu_*x*_PdAu_24–*x*_(2PET)_18_ (*x* = 1, 2, 3) clusters. The incorporation of copper was obtained by reacting the base units with a Cu-2PET complex, thereby substituting individual gold atoms with copper.^79,95^ The UV/vis and MALDI-MS spectra of the Cu_*x*_PdAu_24–*x*_(2PET)_18_ clusters with (*x* = 0–3) are shown in Figures S1 and S4, respectively. However, when the mono-, bi-, and trimetallic clusters were supported on ZnO by solvent evaporation, the desired homogeneous cluster distribution on the ZnO support surface could not be achieved.

As shown by TEM in Figure S5, the nonpolar nature of the 2PET ligand hindered an effective interaction with the polar ZnO surface, leading to cluster agglomeration and formation of ∼25 nm large aggregated cluster islands upon solvent evaporation. Nevertheless, the 2PET ligand still exhibited the desired characteristics in direct synthesis, such high yields and overall cluster stability. Thus, instead of trying other ligands, a partial postsynthetic ligand exchange was pursued to improve the cluster attachment to the ZnO surface.

*p*-Mercaptobenzoic acid (pMBA: −SC_6_H_4_COOH) was thus chosen as an exchange ligand, based on its size similar to 2PET and its carboxylic functional end group, promoting chemical binding to the ZnO surface. A direct synthesis using only pMBA instead of 2PET would lead to smaller clusters with a higher Cu/Au ratio when doped with Cu, as recently reported.^96^ The ligand exchange method is described in detail in the SI. For Au_25_(2PET)_18_ and PdAu_24_(2PET)_18_, where 6–14 and 1–7 ligands, respectively, could successfully be exchanged while maintaining the cluster structure integrity. In contrast, the trimetallic cluster Cu_*x*_PdAu_24–*x*_(2PET)_18_ decomposed upon ligand exchange, and its typical fractions could no longer be detected by MALDI-MS (signal appearing only in the low mass region not relevant for clusters).

To enable the synthesis of Cu_*x*_PdAu_24–*x*_(2PET)_18–*y*_(pMBA)_*y*_, knowing that Cu reduces the cluster stability, the overall sequence had to be reversed. First, the pMBA ligand exchange was performed for the base unit PdAu_24_(2PET)_18_, resulting in PdAu_24_(2PET)_18–*y*_(pMBA)_*y*_ with *y* = 1–7. Second, Cu doping was carried out using the Cu-2PET complex.

The procedure’s success was validated by UV/vis spectroscopy and MALDI-MS. Figure 1 presents the UV/vis spectra, showcasing the sequential steps from the as-prepared PdAu_24_(2PET)_18_ cluster via the ligand exchange and Cu doping to the final trimetallic cluster product. Figure 2 (top) displays the mass spectrum following ligand exchange of PdAu_24_(2PET)_18_, the bottom part characterizes the trimetallic clusters with 0–3 Cu dopants and corresponding ligand distribution. Bimetallic Cu_*x*_Au_25–*x*_(2PET)_18–*y*_(pMBA)_*y*_ clusters were prepared analogously to the trimetallic, with the exception that the synthesis was performed starting from the Au_25_(2PET)_18_ base structure. UV/vis absorption spectra and MALDI-MS of these clusters are shown in Figures S6 and S7, respectively.

**Figure 1:**
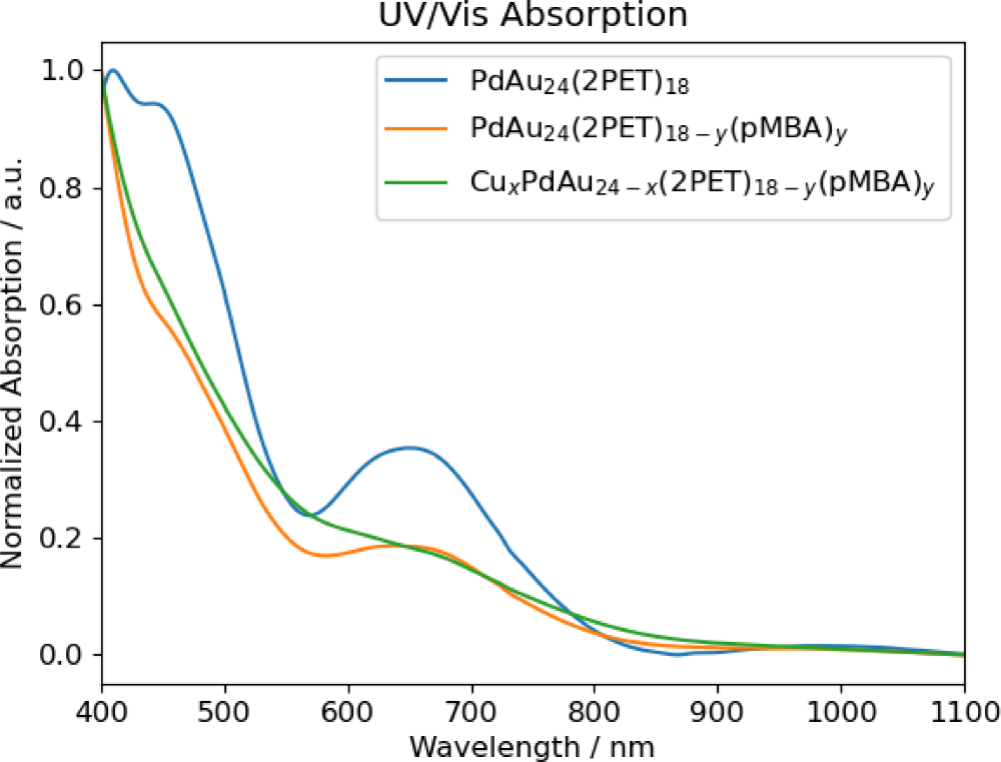
UV/vis absorption spectra of PdAu_24_(2PET)_18_ (blue), after ligand exchange PdAu_24_(2PET)_18–*y*_(pMBA)_*y*_ (*y* = 1–7) (orange) and after doping yielding Cu_*x*_PdAu_24–*x*_(2PET)_18–*y*_(pMBA)_*y*_ (*x* = 0–3, *y* = 0–3) (green).

**Figure 2:**
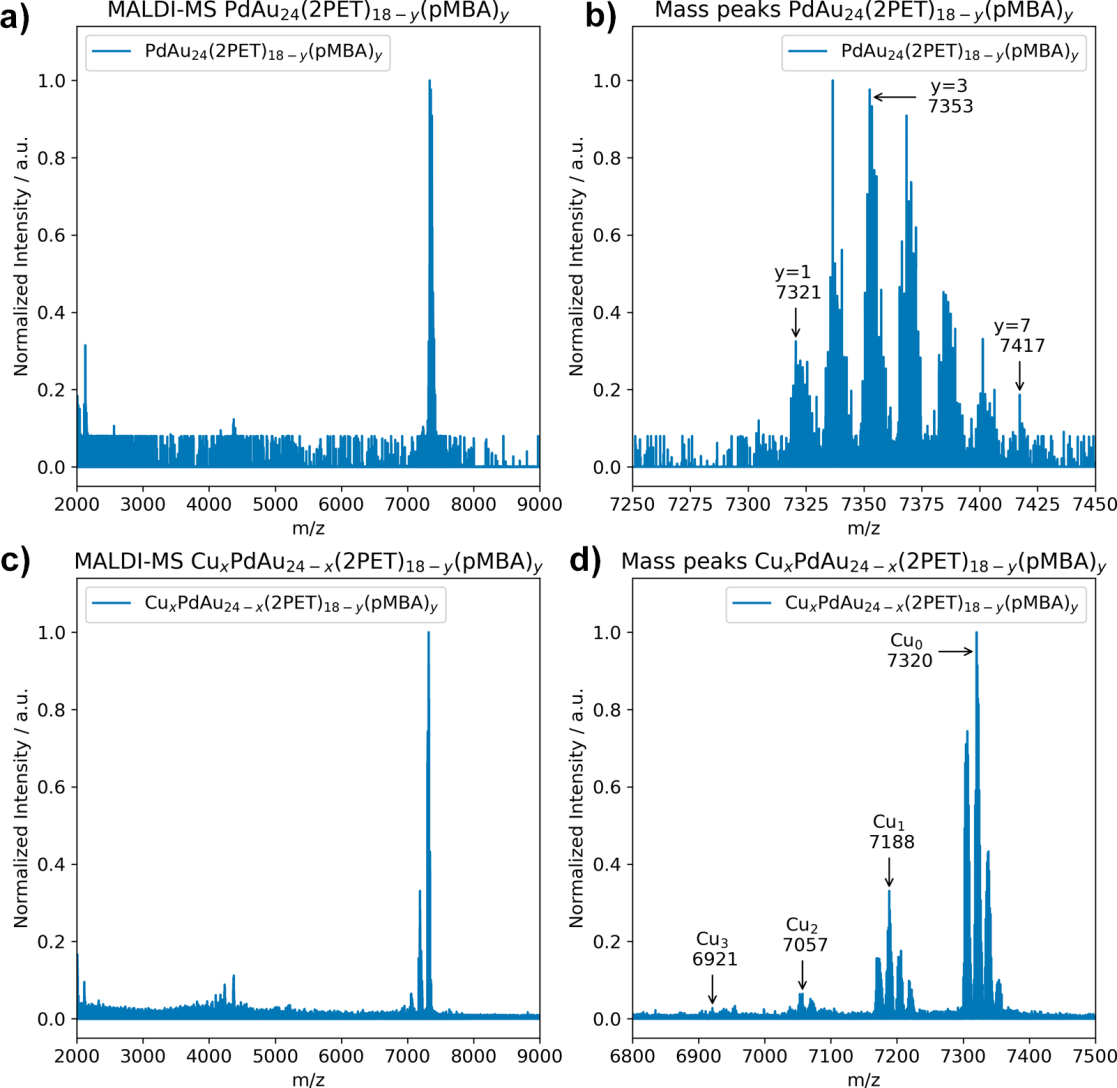
(a) MALDI-MS spectrum of PdAu_24_(2PET)_18–*y*_(pMBA)_*y*_ (*y* = 1–7) after ligand exchange applied to PdAu_24_(2PET)_18_ (full spectrum), (b) mass peaks range of PdAu_24_(2PET)_18–*y*_(pMBA)_*y*_ (*y* = 1–7), (c) MALDI-MS spectrum after Cu doping of PdAu_24_(2PET)_18–*y*_(pMBA)_*y*_ to Cu_*x*_PdAu_24–*x*_(2PET)_18–*y*_(pMBA)_*y*_ (*y* = 1–7) (full spectrum), and (d) mass peaks range of Cu_*x*_PdAu_24–*x*_(2PET)_18–*y*_(pMBA)_*y*_ (*x* = 0–3, *y* = 0–3).

### Optimization of Catalyst Loading and STEM Imaging

Optimal catalyst loading was evaluated for Au_25_(2PET)_18–*y*_(pMBA)_*y*_. Three distinct samples were prepared with targeted cluster loadings of 0.1, 0.5, and 1.0 wt %. Inductively coupled plasma mass spectrometry (ICP-MS) was employed to precisely adjust the concentrations for immobilization and quantify the remaining precursors in the solvent. Among the three samples, the one with 0.5 wt % loading exhibited the highest quality. Observations by STEM revealed a homogeneous cluster distribution on ZnO, with a particle size slightly less than 1 nm (Figure S8).

While a catalyst of comparable quality could be produced for 0.1 wt % loading, a higher loading is preferable. Results for 1 wt % were unsatisfactory, with only ∼80% of clusters immobilized on ZnO (Table S1), and with particles of ∼2 nm in size. Consequently, a catalyst loading of 0.5 wt % was chosen for the remaining study.

Due to the lower stability resulting from ligand exchange and Cu doping, after immobilization of the clusters, total reflection X-ray fluorescence spectroscopy (TXRF) was employed to quantify their loading, rather than ICP-MS. The clusters’ solvent affinity contributed to discrepancies between the actual loading and the targeted 0.5 wt %. Furthermore, for trimetallic clusters, during doping with the Cu-2PET complex, 2PET re-exchanged with pMBA to become pMBA-free for some clusters, which explains the leading feature of each cluster distribution group in the MALDI-MS spectrum in Figure 2 (bottom right). Nevertheless, these clusters did not bind to the ZnO surface anyway and were washed out during rinsing. Still, despite all obstacles, excellent homogeneity, and distribution on the ZnO support was finally obtained both for bimetallic and trimetallic cluster catalysts.

Figure 3 displays the HAADF-STEM images of ZnO-supported Cu_*x*_PdAu_24–*x*_(2PET)_18–*y*_(pMBA)_*y*_ nanoclusters, while images of the other cluster catalysts are shown in Figure S9. Notably, agglomeration was effectively prevented in all cases, and a homogeneous cluster distribution on the ZnO surface was successfully achieved.

**Figure 3:**
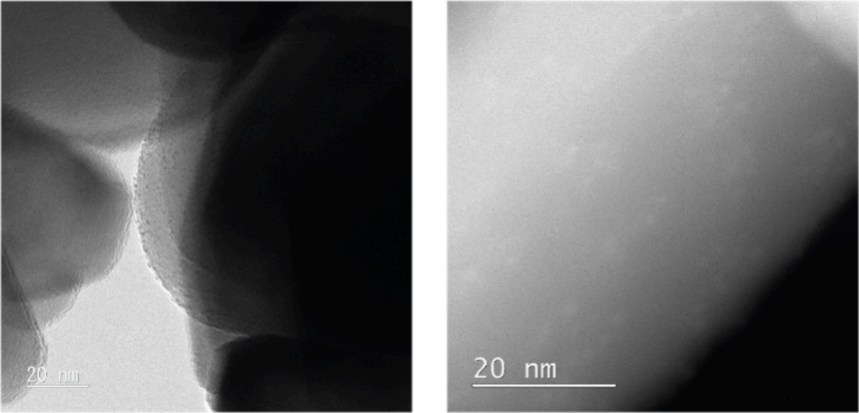
Images of Cu_*x*_PdAu_24–*x*_(2PET)_18–*y*_(pMBA)_*y*_ nanoclusters supported on ZnO. Left: TEM bright field, right: STEM-HAADF

### Catalyst Characterization by XPS and Dopant Quantification

The MALDI-MS results reflect a distribution of unsupported and differently doped nanoclusters and cannot be understood quantitatively, as each cluster has a different ionization potential and stability during measurement. As mentioned before, the introduction of Cu dopants negatively affects the stability of clusters, decreasing it further with each additional Cu atom, leading to a lower signal intensity of its mass peak in the MALDI-MS spectrum. Furthermore, all dopant concentrations were below the detection limit of TXRF. Therefore, XPS was applied after immobilization on the ZnO support (“as-prepared”). Despite the high XPS surface sensitivity, only the high dispersion of the uniformly structured nanoclusters allowed a quantitative analysis of the dopants. In Figure 4, XPS Au 4d and 4f, as well as the most intense dopant regions Cu 2p and Pd 3d are depicted for the as-prepared nanocluster catalysts. Quantification was carried out by normalization to the Zn 2p (ZnO support) and Au 4f region (Table S2), yielding average stoichiometries (ignoring the ligands here) of Cu_2_Au_23_, Pd_1_Au_24_, and Cu_3_Pd_1_Au_21_ of doped nanoclusters, thereby once more confirming the cluster stability during the supporting procedure. Note the detection of even single Pd dopant atoms as a small shoulder on the higher binding energy side of the Au 4d_5/2_ peak despite the low loading (Figure 4a). The “masking” of the Cu 2p_1/2_ peak with the overlapping Al K_β_ satellite of the Zn 2p region (Figure 4b; 0.6% of the Zn 2p intensity at 69.7 eV lower binding energy), the latter being significantly more intense than the Cu signal, also corroborates the low Cu concentration expected from doping by few atoms only.^97^

**Figure 4:**
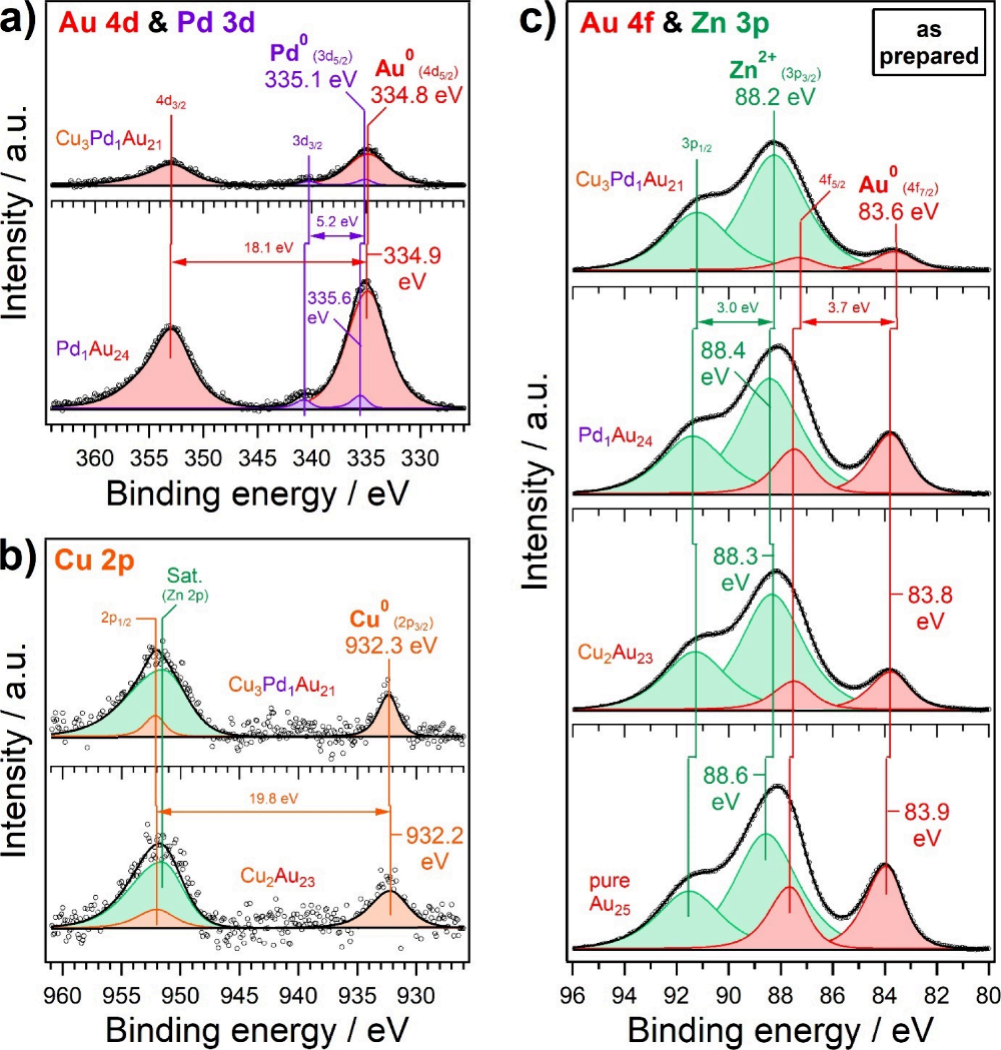
XPS spectra of (a) Au 4d and Pd 3d, (b) Cu 2p, and (c) Au 4f and Zn 3p regions of as-prepared SAA clusters on ZnO support. Au_25_ served as a reference.

The electronic ground state of an undoped nanocluster is primarily determined by the nanosize effect and charge transfer from Au to the ligands, depending on the polarity of the thiol.^98–100^ Upon decreasing size, the cluster core levels shift to higher binding energies due to narrowing of the valence band, which leads to more efficient screening in the final state. This holds true for any supported cluster.^101–103^ In the final state of photoemission, the creation of positively charged core holes further contributes to an upshift in binding energy. Considering the mentioned effects, the Au 4f and 4d binding energies of undoped nanoclusters are expected to be higher or at least comparable to those of *fcc* Au (e.g., Au 4f_7/2_ at 84.0 eV). Indeed, this is confirmed by Figure 4a,c, in agreement with earlier studies.^68,100,104–106^

Doping then caused an additional charge transfer within the cluster, reported as the alloying effect.^95,107–109^ Figure 4c demonstrates that 1–2 dopants per cluster have little to no effect on the Au 4f binding energy, but in the case of trimetallic clusters with more dopants, a pronounced shift to lower binding energy (i.e., −0.3 eV with respect to the undoped Au clusters) was detected. This indicates that cluster doping increased the electron density of Au atoms, which also seems to apply to Pd (Figure 4b) when Cu atoms are present as well.

### Catalyst Activation by Oxidative and Reductive Pretreatment for Ligand Removal

To facilitate the adsorption of WGS reactants on the metal atoms, it is imperative to at least partially remove the ligands from the metal sites by thermal pretreatments.^68^ Initially, the catalysts were exposed to oxidative conditions (21 vol % O_2_ in He, 20 mL/min, 300 °C), which induces ligand removal and also removes carbonaceous species. Subsequently, catalysts were reduced with hydrogen (5 vol % H_2_ in He, 20 mL/min, 300 °C) to obtain metallic clusters. The benchmark catalyst Cu/ZnO/Al_2_O_3_ underwent the same treatment as the supported clusters, respectively single-atom alloy catalysts.

### In Situ DRIFTS of CO Adsorption Confirms Single Atom Sites

To illustrate the successful ligand removal and the accessibility of dopant atoms, in situ DRIFTS spectra of CO adsorption at 35 °C were measured before and after pretreatment (in He and upon addition and removal of CO).^110^ Details of the experimental procedure are described in the SI.

### Before Pretreatment Thiol Ligands Hindered CO Adsorption

Figure S10 shows in situ DRIFTS spectra of CO adsorption before pretreatment. Notably, no peaks of adsorbed CO were observed, as anticipated for cluster catalysts with intact ligand shells. Only the CO gas phase double band was present with its intensity depending on the CO gas pressure. The Cu/ZnO/Al_2_O_3_ reference catalyst also displayed no CO adsorption. On the ZnO support, no discernible CO-ZnO interactions were observed, in line with literature indicating that CO adsorption bands on Zn^2+^ occur within the wavenumber range of 2188–2180 cm^–1^, albeit typically at low temperature (77 K).^111,112^

### Accessibility of Metal Atoms after Pretreatment

CO adsorption spectra after pretreatment are illustrated as “gas-phase-free” in Figure 5, and the original spectra are shown in Figure S11. In Figure 5, the CO gas-phase signal was subtracted from the spectra, allowing for better visibility of the CO adsorption bands. The procedure is described in the SI and illustrated in Figure S12. After O_2_ and H_2_ pretreatment, characteristic CO adsorption peaks were discernible in all samples except the pure ZnO blind test (Figure 5a) and the Au catalyst (Figure 5f). A closer examination of CO adsorption on the technological Cu reference catalyst revealed three overlapping bands at 2125, 2106, and 2090 cm^–1^. These bands are attributed to CO adsorption on distinct binding sites of Cu^+^ or Cu^0^. Unlike conventional carbonyls characterized by distinct σ-donation of CO and π-backbonding from the metal, Cu–CO exhibits a nonclassical behavior, making the differentiation between Cu^+^–CO and Cu^0^–CO more difficult.^113^ The absence of a red shift in the peak of Cu^0^–CO, which appears in the same wavenumber range as Cu^+^–CO, further complicates this distinction.^112,114,115^ Cu^+^–CO exhibits greater stability due to stronger binding (π-repulsion). A direct comparison between the Cu reference and the CuAu SAA in Figure 5b,c hints at different oxidation states. Upon removing CO by purging the cell with He, the CO adsorption IR bands persisted on the Cu reference, whereas on the CuAu SAA catalyst, the single CO adsorption band at 2110 cm^–1^ declined simultaneously with the gas-phase CO peaks, eventually reaching the baseline. This suggests that Cu in the SAA is likely in oxidation state Cu^0^, while in the Cu reference, it is likely Cu^+^. The Cu-CO IR band in the CuAu SAA catalyst in Figures 5c and S11 is quite narrow (FWHM = 24 cm^–1^ (Gauss fit)), suggesting only a linear binding site occupancy. Considering the high signal intensity despite low Cu concentration, it can be assumed that single Cu atoms are located on the clusters’ surface. Although DRIFTS cannot rule out the presence of Cu^2+^, as CO interacts with Cu^2+^ solely through electrostatics or weak π-bonds, the associated adsorption bands in the typical energy range of 2200–2150 cm^–1^ were not observed at 35 °C.

**Figure 5:**
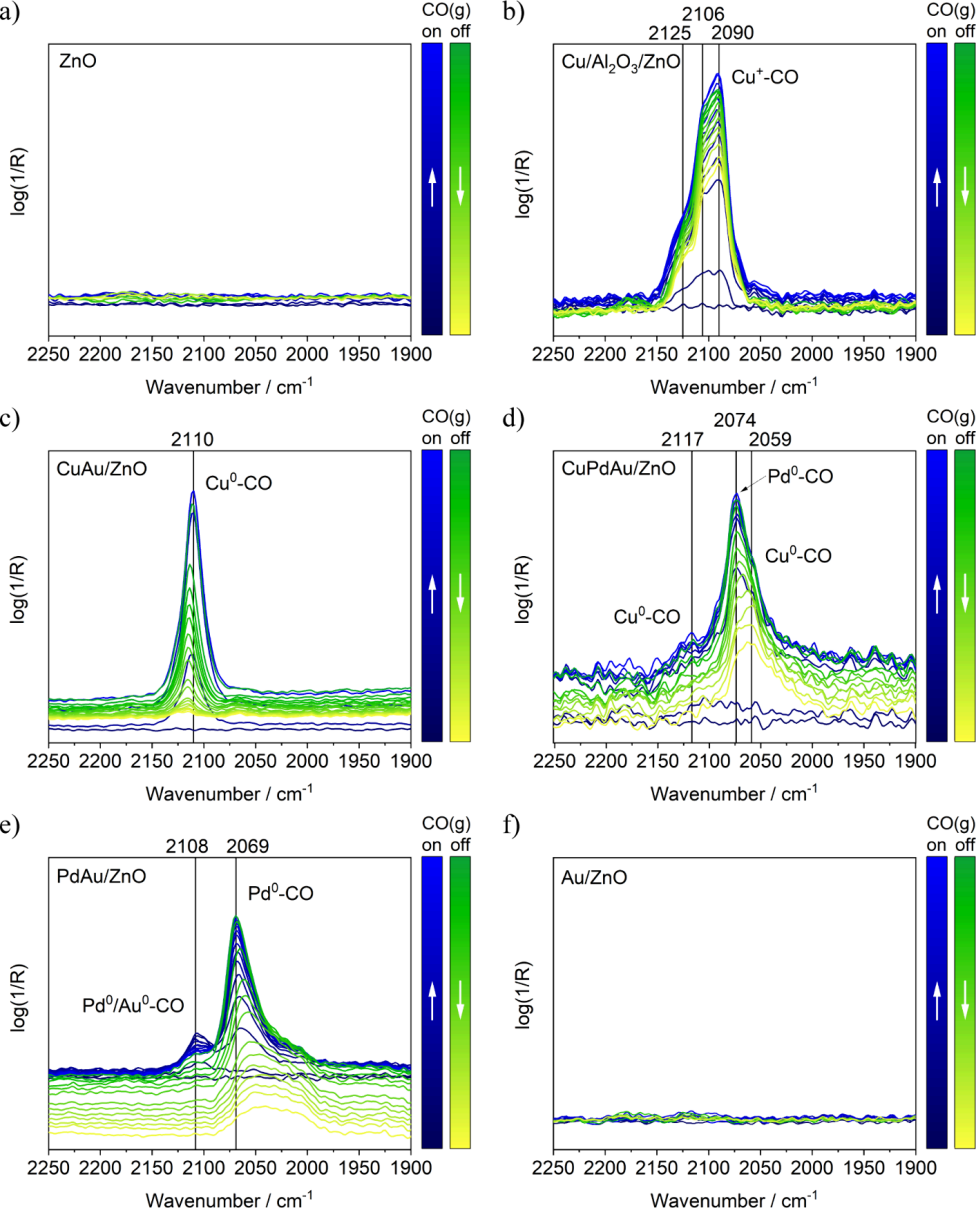
In situ DRIFTS spectra of CO adsorption after pretreatment (blue over green to yellow, *T* = 35 °C, 1 bar, 47.5 mL/min He + 2.5 mL/min CO (added and removed)). The CO­(g) spectrum is subtracted. (a) ZnO blind test only showing baseline. (b) industrial benchmark catalyst Cu/ZnO/Al_2_O_3_ with three binding sites for CO on Cu indicating the Cu^+^ state. (c) CuAu SAA with a single linear adsorption band for CO on Cu. (d) CuPdAu SAA with adsorption features of CO for Cu^0^ and Pd^0^. (e) PdAu SAA showing CO adsorption on Pd^0^ and on Pd^0^/Au^0^ mixed metal site. (f) Au/ZnO not showing CO adsorption.

For the PdAu SAA, two adsorption bands at 2108 and 2069 cm^–1^ occurred in addition to the CO_(g)_ band (Figure 5e). The feature at 2069 cm^–1^ is a linear binding mode of CO on a single Pd surface atom. In the initial cluster structure, Pd is located at the center of the structure. As previously reported, Pd migrates reversibly to the Au surface under oxidative conditions or by the presence of CO.^71,116,117^ This wavenumber agrees with findings of Lear et al., who studied Pd nanoparticles supported on alumina by FTIR, revealing only corner sites for each Pd particle due to small size.^118^ Pd–CO interaction apparently has higher stability than Cu^0^–CO, as adsorption persisted during He purging.

Interestingly, the band at 2108 cm^–1^ resembles CO adsorption on Au in PdAu. Despite the absence of Au^0^–CO bands for the pure Au catalyst, a synergistic effect caused by neighboring single Pd atoms appears sufficient to promote Au–CO adsorption. Abbott et al. reported CO adsorption bands at 2114 and 2086 cm^–1^ for a Au/Pd (1:5) alloy system, which red-shifted to 2107 and 2073 cm^–1^, accompanied by intensity rise when the Au ratio increased.^119^ Due to the SAA catalysts′ high Au to Pd ratio (24:1), a shift to 2108 and 2069 cm^–1^ is reasonable.

For the trimetallic CuPdAu SAA, both bands were again present (Figures 5d and S11) but exhibiting a slight blue shift. The shift may be attributed to a lower Au to Pd ratio (21:1), as copper partially replaced gold, or it may result directly from the influence of copper on the cluster’s electronic structure. At 2117 cm^–1^, a minor feature of atop CO adsorption on Cu^0^ was observed. The trimetallic catalyst, with a lower copper loading than the bimetallic CuAu counterpart, displayed a blue shift of 7 cm^–1^ in the CO–Cu band. The lower adsorption intensity may not only be attributed to trimetallic SAA having the lowest metal loading among all samples, but could be due to its inherent electronic structure affecting CO adsorption.

Thus, the CO-DRIFTS spectra confirmed successful ligand removal through a mild pretreatment: first under oxidative conditions, followed by a reductive step. Linear CO adsorption bands for Cu^0^ and Pd^0^ atoms in the SAAs demonstrate their atomic isolation in the Au host and confirm preparation success. The observed shift or disappearance of the CO adsorption band upon He purging is attributed to the reversible and weak binding of CO on single atoms, potential dopant migration within the clusters, and the dynamic coordination environment under changing gas atmospheres. These factors influence the CO adsorption behavior without contradicting the presence of single-atom sites. Noteworthy, pure Au in the presented state does not interact with CO at room temperature. XPS analysis after pretreatment (vide infra) revealed that surface Au atoms remained partially charged. CO adsorption bands should be observable in DRIFTS, provided that the Au surface is accessible. Two states can explain their absence. First, Au–S remains from the thiol ligands, which survived the pretreatment, still occupying the Au atoms. Additionally, the remaining charged Au sites may be covered by ZnO after ligand removal, again because of SMSI. Both states inhibit interaction between Au and CO. However, the presence of Pd facilitated the reduction of neighboring Au atoms, resulting in additional CO adsorption features characteristic of Au–Pd interactions, as reflected by a weaker additional adsorption band in the CO-DRIFTS spectra. The presence of multiple adsorption sites on Cu was observed for the benchmark catalyst, all of them binding CO stronger than the Cu in the SAAs. This might be related to a higher oxidation state of the benchmark catalyst or differences in electronic structure and binding sites. Nevertheless, the difference in CO interaction could be a key feature of the SAAs in the reaction. Interestingly, slight shifts of the adsorption bands among the catalysts were observed, demonstrating the influence of single atoms on the overall electronic structure of the small clusters.

### XPS Characterization after Pretreatment

Photoelectron spectra acquired after oxidative and reductive pretreatment are depicted in Figure 6, analogous to Figure 4. In comparison to the as-prepared samples, three changes were observed in all regions: (i) a significant shift to lower binding energy, summarized in Table S3, (ii) a decline in signal intensity relative to that of the ZnO support, and (iii) the presence of positively charged cluster atoms. All three changes are consistent, assuming that an oxidative SMSI effect occurs, as previously described for Au/ZnO catalysts.^92,120–122^ In this process, the clusters become at least partially covered with ZnO, which attenuates the signal intensities of the cluster species by increasing the cluster-support interface. The latter promotes charge transfer from the cluster to the support, resulting in positive charging of the cluster shell. Moreover, the ZnO overlayer adds to the cluster size, which lowers the nanosize effect and, in turn, leads to a downward shift in binding energy.

**Figure 6:**
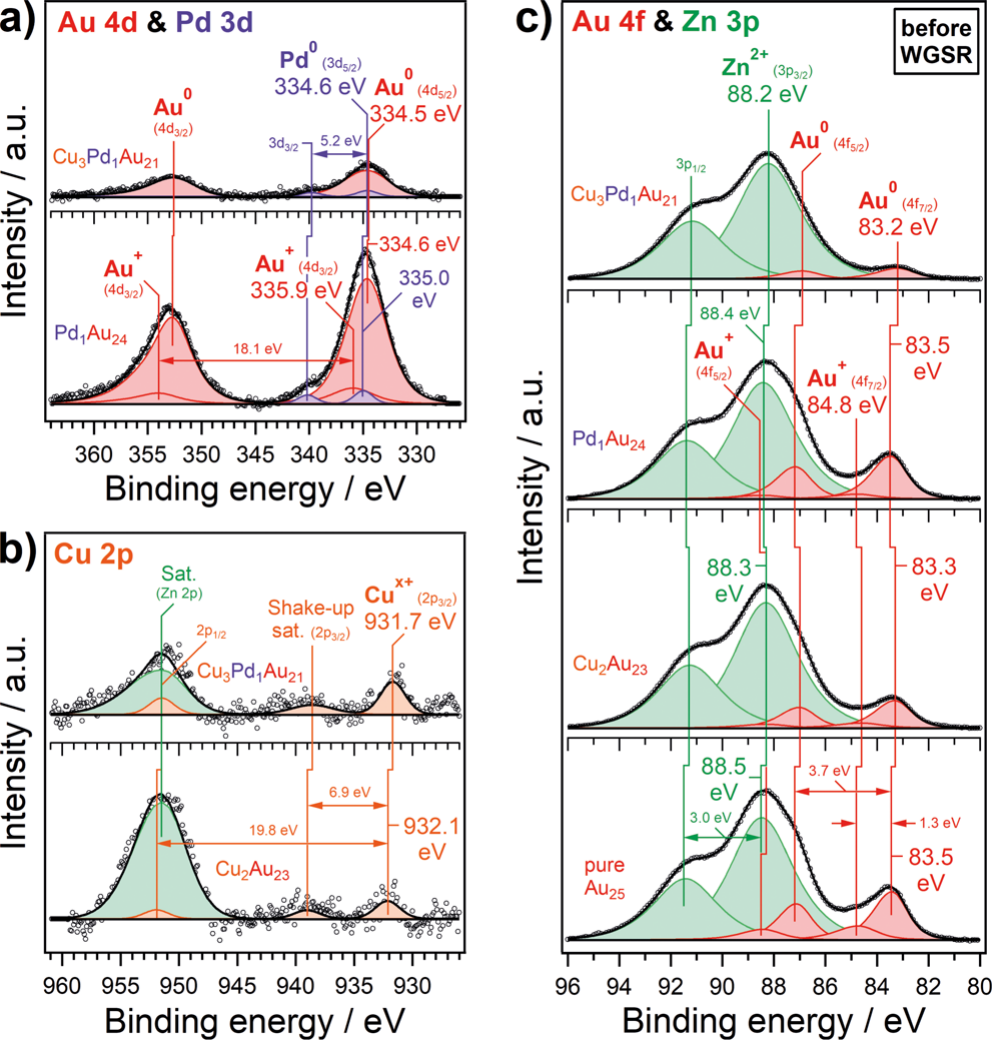
XPS spectra in (a) Au 4d and Pd 3d, (b) Cu 2p, and (c) Au 4f and Zn 3p regions of (doped) SAA catalysts on ZnO support, acquired after pretreatment. Au_25_ served as a reference.

In the case of Pd, the shift to lower binding energy (PdAu_24_: −0.6 eV; Cu_3_PdAu_21_: −0.5 eV) may also be related to lower coordination when Pd migrates from the cluster center to its surface during pretreatment (Figure 6).^123^ The Pd migration from the gold cluster’s center to its surface is expected based on detailed examination in previous studies.^71,124,125^ According to DRIFTS, Pd remains uncharged, though. Shake-up satellites in the Cu 2p region primarily indicate the presence of Cu^2+^ (Figure 6b). However, Cu^+^ and Cu^0^ oxidation states (the latter was observed by DRIFTS) may also contribute to the main peaks, thus labeled “Cu^*x*+^”. Au^+^ binding energy positions in the Au 4f (Figure 6c) and 4d regions agree well with those determined by Buitendach et al. for Au­(I) complexes and the Au^+^/Au^0^ ratio decreases in the order Au_25_ > Cu_2_Au_23_ > PdAu_24_.

A decrease in XPS intensity is also often associated with cluster agglomeration, forming nanoparticles.^126^ Accordingly, one would expect that the dopant intensities are the first to vanish (especially those of Pd), and a shift to higher binding energy toward the respective “metallic state” of cluster atoms should occur. Given that the opposite was observed, cluster agglomeration can be largely excluded, which is corroborated by the (S)­TEM results discussed later.

### WGS Catalysis of SAA vs the Benchmark Cu/ZnO/Al_2_O_3_ Catalyst

For a meaningful comparison of catalysts, the kinetic results from WGS flow reactor tests were normalized to a nominal 1 wt % metal (Cu, Pd, and/or Au) loading relative to the support (as determined by TXRF). The loadings and relevant factors are listed in Table S5. Figure 7 compares catalytic activity in terms of CO conversion and H_2_ formation for all catalysts over two consecutive runs from 250 to 400 °C. The H_2_ signals shown in Figure 7b,d are intended for qualitative comparison only, reflecting the same trend as CO conversion. Below 250 °C, no activity was observed. In the first run, the order in activity at 400 °C was: ZnO < Au < Cu-ref < PdAu = CuAu < CuPdAu.

**Figure 7:**
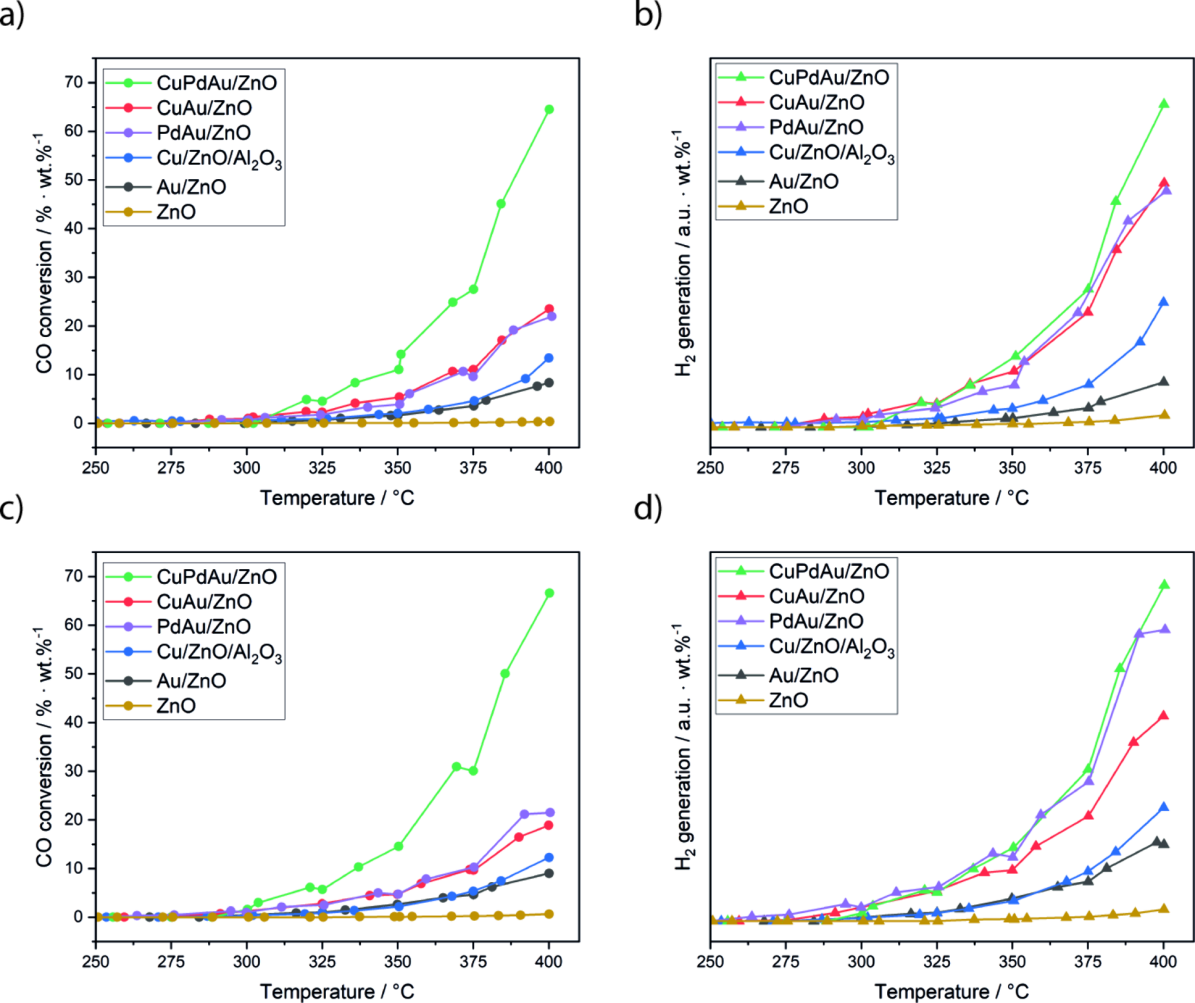
CO conversion and hydrogen production during WGS reaction normalized to the catalyst loading (1 wt %). (a) CO conversion during the first WGS reaction cycle, (b) corresponding H_2_ generation for the first cycle, (c) CO conversion during the second WGS reaction cycle, and (d) corresponding H_2_ generation. In between runs, the catalysts were cooled to room temperature. In Tables S6–S8, space-time conversion (STC), space-time yield (STY), and turnover frequencies (TOF) are reported.

The two cycles are indicative of catalyst stability, with SAA catalysts exhibiting even higher activity in the second run, except for CuAu SAA, whereas the benchmark catalyst showed a small decrease in hydrogen production. Although the catalytic results must be interpreted with caution, as transport and diffusion limitations are not accounted for in the normalization, the significant enhancement in intrinsic activity due to dopant incorporation remains evident. These results underscore the superior activity of cluster-based single-atom-alloy catalysts, showcasing their potential as robust alternatives in catalytic applications.

### Postreaction XPS Characterization

The XPS regions displayed in Figures 4 and 6 were also re-examined after carrying out WGS reaction (Figure 8). While the signal intensities of cluster species increased slightly, the trend toward lower binding energies continued, especially for Cu_2_Au_23_ (Cu 2p: −0.9 eV) and PdAu_24_ (Pd 3d: −0.6 eV), as expected for SAA catalysts. Furthermore, the charge neutrality of Au and Cu was restored. This suggests that the SMSI effect became less pronounced, and clusters were consequently less covered with ZnO (partial reversal). The final peak positions are clearly lower than those of *fcc* Au, Cu (e.g., Cu 2p_3/2_ at 932.6 eV), and Pd (e.g., Pd 3d_5/2_ at 335.4 eV). Binding energy shifts relative to the pretreated state and compositional changes compared to the as-prepared state are summarized in Tables S3 and S4, respectively.

**Figure 8:**
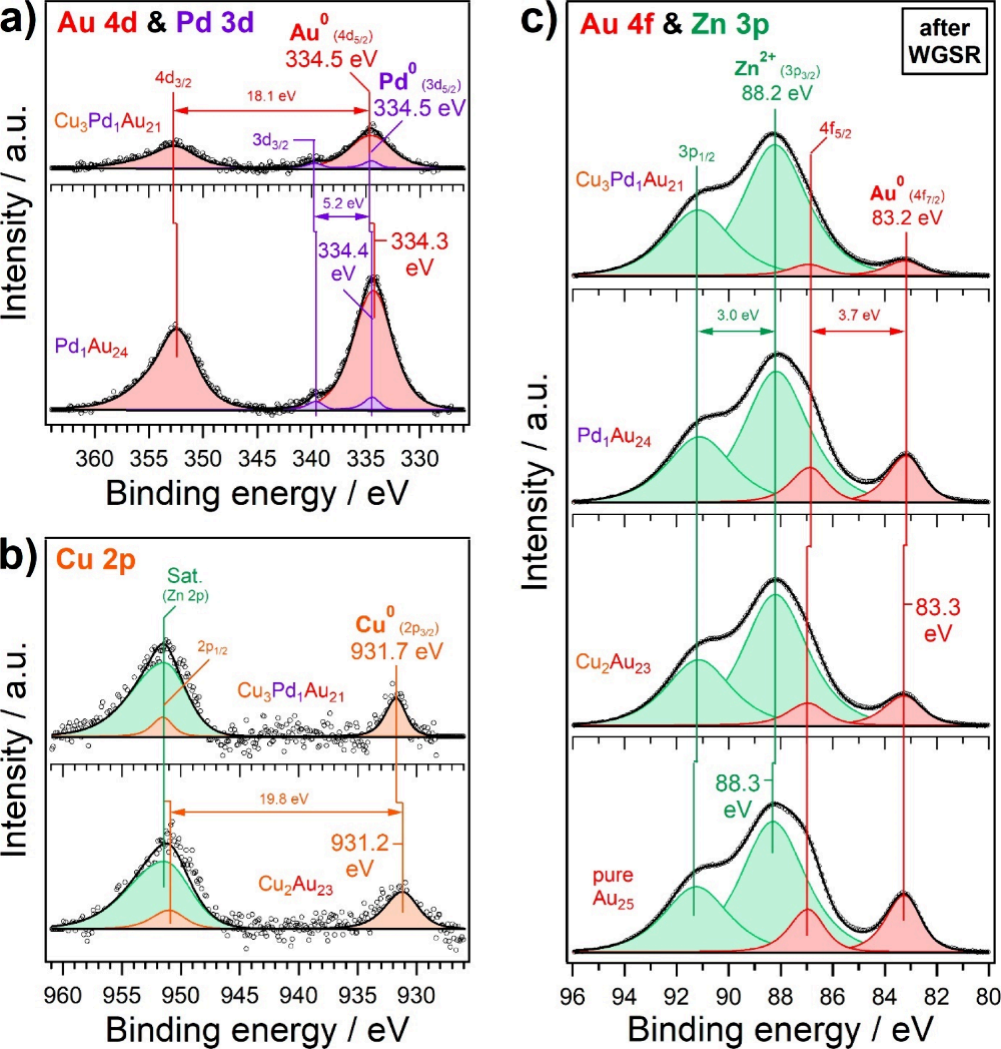
XPS spectra of (a) Cu 2p, (b) Au 4d and Pd 3d, and (c) Au 4f and Zn 3p regions of (doped) SAA clusters on ZnO support, acquired after pretreatment and WGS. Au_25_ served as reference.

### Catalyst Evolution and Fate of the Thiol Ligands

(S)­TEM measurements were performed of the SAA catalysts as synthesized, after pretreatment, and after WGS. The images are shown in Figure 3 and in the SI in Figures S9, S13, and S14. The particle size analysis is summarized in Table S9. For monometallic Au and bimetallic PdAu, the average particle diameter increases from 1.0 ± 0.3 nm (as-prepared) to 2.2 ± 0.8 nm and from 1.0 ± 0.2 to 2.2 ± 0.9 nm, respectively, after two reaction cycles of WGS reaction. While this reflects some degree of particle coalescence, the absolute size remains small, suggesting that no extensive sintering or large-scale agglomeration has occurred. Notably, when Cu is present, the alloyed systems exhibit even more restrained changes, highlighting the beneficial role of alloying in the structural stabilization. The CuAu system grows moderately, from 1.1 ± 0.3 to 1.8 ± 1.0 nm. Notably, the trimetallic CuPdAu exhibits the smallest change, increasing from 0.9 ± 0.2 to 1.7 ± 0.8 nm. The consistently narrow standard deviations further support the presence of well-dispersed particles with uniform size distribution. The stability of the single-atom catalysts during WGS is still astonishing. Typically, temperatures exceeding 300 °C are avoided to prevent cluster agglomeration to larger nanoparticles, which typically decreases activity. In our study, the combination of XPS and DRIFTS has already demonstrated an SMSI effect after pretreatment that covers at least part of the clusters with the support material while still allowing reactant adsorption. This seems to stabilize the particles. However, once the ligands are removed, (S)­TEM imaging becomes difficult, as electron irradiation restructures the particles and significantly accelerates the mobility of ZnO, hence strongly inducing further SMSI. Accordingly, the images in Figures S13 and S14 were acquired with minimal possible electron beam dose. Due to the small cluster size, most surface sites occupied by the dopants can be considered low-coordination sites, which appears to be beneficial for the reaction, explaining the unexpected stability and performance of the SAA cluster catalysts for WGS.

STEM images of the industrial-grade Cu reference catalyst, as-prepared and in its post-reaction state, are shown in Figure S15. The structural evolution of the Cu benchmark sample was different from that of the SAAs. In the as-prepared state, unlike the clusters, the Cu particles have a size distribution ranging from 4 to 40 nm. However, after the reaction, Cu was scarcely observed by STEM, and no SMSI effect was detected for the benchmark. This observation can be rationalized by adsorbate (CO)-induced redispersion of Cu, leading to the formation of single-atom Cu species on ZnO, which are below the STEM detection limit.^113^ Nonetheless, a few particles, ranging from 3.7 to 16.8 nm, were still found after the reaction. Considering that the reaction can take place on single Cu sites, the higher activity of the cluster-based bimetallic and trimetallic catalysts must be due to a synergistic effect of Cu with Au and/or Pd, despite the significantly lower Cu content and the low activity of the pure Au catalyst.

In situ DRIFTS measurements were performed throughout the entire reaction sequence, starting with the as-prepared sample, monitoring the pretreatment steps, and the two catalytic runs. Figure S16 shows the corresponding spectra of the trimetallic SAA, all acquired at 40 °C for better comparison. Figure S16a displays the region from 3800 to 3100 cm^–1^ with the interpretation of the hydroxyl groups based on the findings of Noei et al.^127^ The as-prepared sample (black) showed two bands at 3640 and 3623 cm^–1^, which represent hydroxyl groups on the ( $10\overset{®}{1}0$ ) and ( $000\overset{®}{1}$ ) ZnO surfaces, which strongly decreased upon oxygen pretreatment. However, another surface OH signal appeared at 3680 cm^–1^, indicating a restructuring of the ZnO surface. After the first reaction run (dark green), a band showed up at 3695 cm^–1^, i.e., H_2_O coadsorbed with hydroxyl groups. The H_2_O apparently originated from the reaction gas mix, which kept flowing between the first and second runs. Only after the second reaction run (light green) the feed gas was changed to pure He, removing H_2_O from the system. The band at 3680 cm^–1^ then seemed to have slightly increased, meaning the number of surface OH groups on ZnO increased as compared to before the reaction, and another hydroxyl signal at 3573 cm^–1^ related to ZnO defect sites emerged.^128^ The formation of defect sites originates from the reaction and is likely connected to the observed SMSI effect. For an encapsulating SMSI, usually a metal with high surface energy, e.g., Pt or Pd, together with a metal oxide with low surface energy, is a prerequisite, making Au supported on ZnO not an obvious candidate.^129^ However, the surface energy of gold increases strongly with decreasing particle size.^130^ Furthermore, defects, adsorbates, and hydroxyl surface groups lower the surface energy of ZnO.^131^ Among different reactions, the Au/ZnO SMSI was studied in detail for methanol synthesis, for which a reductive medium of CO_2_ and H_2_ is present. Further tests by Wiese et al. comparing both CO_2_:H_2_ and CO:H_2_ mixtures revealed an even stronger SMSI effect when the latter was applied.^132^ Since all three components, i.e., CO, CO_2_, and H_2_, were present during WGS, a similar SMSI mechanism can be assumed: Reduction of ZnO by H_2_, with CO producing mobile Zn species and oxygen vacancies. Zn then migrates onto Au, where it gets reoxidized, likely by CO_2_, covering the clusters’ surfaces and completely encapsulating Au with time.^132^ WGS blind tests of pure ZnO (after the same pretreatments) revealed small H_2_ production starting at 300 °C. More relevant for SMSI is that CO_2_ can be detected already in the temperature range from 225 to 240 °C. However, the formation of a significant number of oxygen vacancies in the ZnO support was reported at 240 °C by the reaction of CO and ZnO to CO_2_ and ZnO_*x*_.^132^ CO_2_ formation occurred similarly for the SAAs, for which the onset temperature of support reductions seems unaffected.

SMSIs have three effects on the structures: (i) a geometric effect, here denoted by the encapsulating layer, (ii) a bifunctional effect, and (iii) electron transfer between the metal and the support. The bifunctional effect manifests by a new reaction site formed at the boundary between metal and support material, which differs from the rest of the metal or support and can positively affect catalytic activity.^89,133–136^ Such Au–ZnO_*x*_ (0 ≤ *x* < 1) sites might be the active phase in the Au catalyst, explaining the increased activity in the second run compared to the first. Analogously, Bollmann et al. reported higher WGS activity observed for Pd nanoparticles when alloyed with Zn.^137^

The presence and influence of a AuZn alloy phase could neither be confirmed nor excluded, as the dominant ZnO signal in the XPS spectra obscures potential Zn alloy-related features. Nevertheless, Pd and Cu dopants have a markedly stronger impact on catalytic activity, with the resulting single-atom alloys demonstrating significantly improved performance relative to both the undoped Au/ZnO system and the industrial Cu/ZnO/Al_2_O_3_ benchmark catalyst. Despite the positively charged Au surface, demonstrated by XPS, CO–Au^+^ adsorption was inhibited and not observed in DRIFTS, likely due to the occupancy of Au by remaining S and a partial ZnO cover, both a direct result of the SMSI. Abdel-Mageed et al. started to observe the Au–CO adsorption bands at pressures of 5 bar and higher.^138^ Accordingly, the stronger adsorption of CO to the dopant atoms is responsible for the higher WGS rate for two reasons: First, the dopants bind and provide the reactant CO to the WGS reaction. Second, CO counteracts SMSI by cleaning the SAA’s surfaces from ZnO via CO reduction, leading to decreased SMSI after the reaction, as shown by XPS. In Figure S16c, in the range from 2400 to 1700 cm^–1^, merely gas phase CO at 2175 and 2120 cm^–1^ (dark green) was observed. In Figure S16d typical surface carbonate-like species appeared, formed by adsorption or reoxidation of ZnO_*x*_ (*x* < 2) species on the ZnO surface by CO_2_.

The Cu reference catalyst showed quite a different behavior, with small quantities of CO_2_ already observed at 50 °C in agreement with previous studies.^10^ In this case, CuO_*x*_ is more likely to provide oxygen than ZnO. Furthermore, a restructuring of Cu occurred, as depicted by the different reaction profiles of the first and second runs. In the first, at 200 °C, H_2_ formed at a much lower temperature than for the other samples but did not steadily increase with temperature. The activity remained relatively constant until 275 °C, when the curve adopted the typical temperature-dependent shape. Noteworthy, the onset temperature of H_2_ production in the second run was only at 275 °C as well. Restructuring of the Cu particles occurred under reaction conditions, changing the ability to interact with CO. As discussed above, DRIFTS revealed multiple CO binding configurations after pretreatment. However, after the reaction, Cu seemed to have spread on the catalyst’s surface, creating single atoms or particles below the detection limit of TEM.^113,139^ Although these single Cu atoms/small particle catalysts are industrial state of the art, they are inferior to the SAAs under the current conditions at the atomic level.

Concerning the thiol ligands, Figure S16b depicts the range from 3200 to 2400 cm^–1^. The as-prepared trimetallic sample showed a prominent feature with four peaks between 3020 and 2815 cm^–1^. This overlaps with bands of the solvent tetrahydrofuran (THF), used during immobilization and still adsorbed on the surface, and of the 2PET ligand (3100–3040 cm^–1^, showing the aromatic contribution of the ligands). After the oxidative pretreatment, the signal significantly decreased. THF was removed from the system, whereas 2PET ligands partially remained. Noteworthy, the contribution of 2PET to the spectra can be observed in each experimental step, i.e., some ligands remained intact. Apparently, the observed SMSI effect with ZnO not only formed a protective shell on the clusters, but also prevented complete ligand removal. pMBA seems to be below the detection limit: for trimetallic clusters, based on MALDI-MS, a maximum of 3 pMBA ligands was achieved.

The XPS S 2p regions in Figure S17 demonstrate the initial, pretreated, and final state of the SAA catalysts. In Figure S17a, the ligands are assumed to be fully intact (“Au-SR”; 162.0–162.5 eV), while Figure S17c shows that there are only traces of sulfidic sulfur left on the ZnO support, which may have filled oxygen vacancies in the defect structure of ZnO, forming ZnS (161.3/161.4 eV). Constraining the peak positions and FWHM values from Figure S17a,c, both ligand and ZnS signals were fitted in Figure S17b. ZnO interband transitions due to core-hole creation cause a satellite peak at 16–19 eV higher binding energy than Zn 3s, also reflected by a low binding energy shoulder in the S 2p region.^140–142^ The XPS spectra recorded after pretreatment (Figure S17b) illustrate ligand migration to the support, where they are eventually decomposed. According to previous studies, this process is expected to take place during the oxidative pretreatment step, in which only sulfates from the ligands remain on the support.^68,69,71,143^ The second reductive pretreatment step leads to the conversion of sulfates to ZnS, as shown in Figure S17b. However, except for the trimetallic cluster sample, the ligands appear to have been only partially removed even after reductive pretreatment. The partial ZnO coverage resulting from the SMSI effect likely immobilizes the ligands and protects them from decomposition. Due to the reductive reaction conditions (and reaction temperature), the transformation to ZnS is completed as the ZnO overlayer is reduced. While sulfates and sulfides are formed in comparable quantities for the pure Au_25_ cluster upon pretreatment, more sulfates are present for the Cu_2_Au_23_ catalyst. As shown in the Cu 2p region spectra (Figure S17b), Cu is positively charged and thus appears to support oxidation, but in contrast to Pd it does not support reduction, as more ZnS is associated with the PdAu_24_ catalyst.

## Conclusions

Bi- and trimetallic single-atom-alloy (SAA) catalysts featuring Au_25–*x*_ (*x* = 0–4) clusters as hosts, that were doped by individual Pd and/or few (1–3) Cu atoms, supported on a ZnO support, were successfully synthesized through atomically controlled doping and partial ligand exchange facilitating stable immobilization. The developed synthesis protocol features Cu introduction after ligand exchange, which allowed the catalyst to overcome stability barriers and to achieve the desired polymetallic compositions.

MALDI-MS analysis and immobilization studies by (S)­TEM and XPS revealed that the ZnO supported clusters were approximately 1 nm in size, and with the targeted average metal ratios of Cu_2_Au_23_, Pd_1_Au_24_, and Cu_3_Pd_1_Au_21_.

Subsequently, the SAA catalysts were applied in the water–gas shift reaction and compared to the undoped Au/ZnO catalyst and an industrial-grade benchmark of Cu/ZnO/Al_2_O_3_. The accessibility of the reactants to the single-atom Cu and Pd sites on the cluster surface was confirmed by in situ DRIFTS of CO adsorption. Furthermore, a significant strong metal–support interaction (SMSI) (partial decoration) effect was observed, which stabilized the SAAs against agglomeration, ensuring persistent catalytic activity.

The incorporation of Cu and Pd single-atom sites has a profound impact on catalytic activity, likely due to the resulting stronger CO interactions, which counteract the SMSI effect, thereby exposing active sites on the particles’ surfaces. Furthermore, XPS analysis revealed that, after pretreatment, Cu adopts a positive oxidation state while Pd remains in the metallic state. Additionally, Au atoms in the PdAu cluster appear more reduced compared to Au in Pd-free SAAs. CO adsorption DRIFTS further supports the presence of a Pd/Au alloy phase. Together, these results suggest that Pd facilitates the reduction step, while Cu promotes reoxidation, allowing for a highly efficient catalytic cycle in the trimetallic system.

The (partial) encapsulation also led to a partial preservation of cluster ligands, as confirmed by in situ DRIFTS and corroborated by post-reaction XPS. In the future, this may be utilized to influence selectivity in specific reactions by strategically stabilizing different ligands. It furthermore underscores the potential of polymetallic thiolate-protected nanoclusters as precursors for SAA catalysis.

## Methods

Additional details on the experiments and synthesis procedures are available in the Supporting Information.

### Cluster Synthesis

Au_25_(2PET)_18_ was synthesized through a modified procedure based on a previously reported methodology.^77^ The synthesis of PdAu_24_(2PET)_18_ was performed as described by Takano et al.^61^ Unlike in the original report, the PdAu_24_(2PET)_18_ cluster isolation was performed with a SiO_2_ chromatography column instead of Al_2_O_3_.

### Ligand Exchange

Aliquots of 3 mg cluster were dissolved in 1 mL acetone, and 3 mg of pMBA ligand was added. After sonication for approximately 5 s, the reaction mixture was left at room temperature without stirring for 2 h. Acetone was removed by rotary evaporation, and the precipitant was washed five times with a water–methanol mix (3:7). The suspension was centrifuged, and the aqueous phase was removed by decantation, followed by cluster extraction using acetone. The procedure was repeated twice, and MALDI-MS was used to monitor the degree of ligand exchange.

### Cluster Synthesis Product Characterization

Synthesis success was confirmed by Matrix-Assisted Laser Desorption/Ionization Mass Spectrometry (MALDI-MS) using a JEOL JMSS3000 spectrometer with trans-2-[3-(4-*tert*-butylphenyl)-2-methyl-2-propenylidene] malononitrile (DCTB) matrix in ion positive or negative mode, giving the exact cluster masses. Additionally, the UV/vis fingerprint spectra were taken on a JASCO V-670 UV/vis absorption spectrometer in solution (toluene, dichloromethane, tetrahydrofuran).

### Synthesis of Cu/ZnO/Al_2_O_3_ WGS Reaction Benchmark Catalyst

The zincian malachite precursor with a molar Cu:Zn:Al ratio of 68:29:3 was synthesized by coprecipitation (*T* = 338 K) from a Cu, Zn, Al nitrate solution (1.0 M metal-based) and Na_2_CO_3_ solution (1.6 M) as precipitating agent in an automated lab reactor (OptiMax, Mettler Toledo) at constant pH of 6.5. The precipitate was aged in the mother liquor, filtered, washed, and dried. Calcination was carried out in static air for 3h, with a heating rate of 2 K min^–1^ and up to 623 K. The catalyst meets the specifications outlined in the FHI standard, ensuring optimal performance and reliability for our intended applications.^16^

### Catalyst Characterization

Catalyst cluster loading was quantified using an ATOMIKA 8030C X-ray fluorescence analyzer in total reflection geometry (TXRF) equipped with an energy-dispersive Si­(Li)-detector. Excitation was performed with monochromatized Mo K_α_ (17.48 keV) radiation. The elemental proportions were referenced to 100% Zn during measurement. Catalysts (S)­TEM imaging was performed using a JEM-2100 electron microscope (JEOL) and a FEI Tecnai F20 S-TWIN analytical (S)­TEM, which was equipped with a Gatan GIF Tridiem filter. Both microscopes were operating at 200 kV. X-ray photoelectron spectroscopy (XPS) measurements were carried out within a UHV system (base pressure: 5.0 × 10^–10^ mbar) equipped with a Phoibos 100 hemispherical analyzer and XR 50 X-ray source (SPECS GmbH). XPS measurements were performed in normal emission at room temperature applying Al K_α_ radiation (1486.61 eV), a step size of 0.1 eV, a dwell time of 0.5 s, and a pass energy of 20 eV with the energy analyzer operating in “large area” transmission mode. All spectra were referenced to the C 1s signal (C–C, 284.6 eV).

### Catalyst Pretreatment

50 mg per sample was put under a total flow of 20 mL/min of 21 vol % O_2_ in inert gas. Once equilibrium was reached and controlled with a mass spectrometer, the temperature was increased with a 10 °C/min ramp to the maximum temperature of 300 °C. The sample was kept at 300 °C for 30 min, followed by a cooldown to 35 °C. During cooling, the gases were switched to pure inert gas. Once 35 °C was reached, a flow of 20 mL/min of 5 vol % H_2_ was set. The temperature program was repeated under the reductive conditions.

### Catalytic Activity Tests

Two consecutive catalytic runs in the WGS reaction were performed with each catalyst. After the oxidative and reductive pretreatments described above were completed, the gas mix was changed to the reaction mix at 25 °C (0.75 mL/min CO and 0.25 mL/min H_2_O, 19 mL/min He (total)). H_2_O was added by flowing 9.25 mL/min He through a bubbler kept at 25 °C with a cryostat. The reactor temperature increased by 10 °C/min between 25 and 250 °C, then the heating rate decreased to 5 °C/min up to the maximum temperature of 400 °C. At 50 °C intervals (50, 100, 150, ..., 400 °C), the temperature was kept constant for 5 min to obtain (MicroGC) data points. In the cool down to room temperature between the two consecutive runs, the samples were kept under the reaction gas. After the second run, the samples were cooled under inert gas. Gas chromatography (GC) was performed with a MicroGC Fusion 3000A setup from Inficon. For the separation of H_2_, O_2_, N_2_, CH_4_, and CO, a Molsieve column with 5 Å pore diameter was used. CO_2_ was detected on an RT-Q bond column, and both columns detected the gas with a TCD detector.

### In Situ DRIFTS

DRIFTS studies were conducted using a Bruker Vertex 70 spectrometer equipped with a liquid nitrogen-cooled MCT (Mercury Cadmium Telluride) detector, offering a spectral resolution of 4 cm^–1^. An average of 256 scans were taken during the measurements to ensure a robust signal-to-noise ratio. The experimental setup involved a stainless-steel flow cell (Pike) equipped with a CaF_2_ window. Inside, the sample was set in a cool and heat able crucible, which allows gas permeation. The cell inlet was connected to a gas manifold system featuring calibrated mass flow controllers to adjust the gas mixtures used in the experiments precisely.

CO adsorption experiments were carried out at 35 °C to ensure sample comparability. Initially, the specimens were kept under a continuous He flow at a 47.5 mL/min rate. Infrared spectra were acquired at 3 min intervals. After collecting at least two spectra in the inert gas environment, CO was introduced at a flow rate of 2.5 mL/min until equilibrium was achieved, and the spectra reached a consistent profile. Subsequently, the CO valve was closed, and the gas mixture gradually transitioned back to pure He. The gas content was followed by mass spectrometry.

## Supplementary Material



## Notes

Description of synthesis procedures of clusters and final SAAs; description of characterization methods and procedures; additional plots of UV/vis spectroscopy, MALDI-MS, IR, XPS, activity experiments, and (S)­TEM images to support the main manuscript; and calculations of STC, STY, and TOF (PDF)
