# Supplementary material for: Engineering Catalytic Efficiency by Thiolate-Protected Trimetallic (Cu, Pd, Au) Nanoclusters: Single-Atom Alloy Catalysts for Water–Gas Shift
Source: ACS Catal. 2025 Aug 22;15(17):15459–74. doi: 10.1021/acscatal.5c04165 (PMC12418311; doi:10.1021/acscatal.5c04165)
Supplement: Supplementary file 1 [file cs5c04165_si_001.pdf]

Supporting Information

Engineering Catalytic Efficiency by Thiolate-Protected Trimetallic (Cu, Pd, Au) Nanoclusters: Single-Atom Alloy Catalysts for Water-Gas Shift

*[a)]Stephan Pollitt, [a)]Thomas Haunold, [b)]Sakiat Hossain, [c)]Gereon Behrendt, [d)]Michael Stöger-Pollach, [b)]Tokuhisa Kawawaki, [a)]Noelia Barrabés, [c)]Malte Behrens, [b)]Yuichi Negishi, and [a)]Günther Rupprechter**

[a)]Institute of Materials Chemistry, TU Wien, Getreidemarkt 9/BC, 1060 Vienna, Austria

[b)]Department of Applied Chemistry, Faculty of Science, Tokyo University of Science, Kagurazaka, Shinjuku-ku, Tokyo 162-8601, Japan

[c)]Institute of Inorganic Chemistry, Solid State Chemistry and Catalysis, Kiel University, Max-Eyth-Str. 2, 24118 Kiel, Germany

[d)]University Service Center for Transmission Electron Microscopy (USTEM), TU Wien, Stadionallee 2/057-02, 1020 Vienna, Austria

Corresponding Author

Günther Rupprechter

guenther.rupprechter@tuwien.ac.at

Cluster Synthesis

Synthesis of $Au_{25}(2PET)_{18}$

$Au_{25}(2PET)_{18}$ was synthesized through a modified procedure based on a previously reported methodology.[1] Initially, 54.25 ml of a 30 mM aqueous $HAuCl_4$ solution was evaporated to dryness, yielding $HAuCl_4$ crystals. These crystals were combined with 62.5 ml of tetrahydrofuran (THF) and 900 mg of tetraoctylammonium bromide (TOABr). After 30 min of stirring, 1312 µl of the 2-phenyl ethanethiol (2-PET) ligand were introduced, and the reaction mixture was stirred for an additional 30 minutes.

Subsequently, the reaction solution underwent reduction by adding 712.5 mg of $NaBH_4$ dissolved in 12.5 ml of H_2O at 0°C, with vigorous stirring maintained for 14 hours. Termination of the reaction was achieved by THF removal. A water-methanol mixture cleaning procedure (H_2O:MeOH=1:4) was implemented. Following water removal through centrifugation and decantation, the resulting cluster mix was extracted with toluene.

Upon complete toluene evaporation, the $Au_{25}(2PET)_{18}$ clusters were further extracted using an acetonitrile-acetone mixture (C_2H_3N:C_3H_6O=4:1). The product purity was ascertained through UV/Vis spectroscopy and Matrix-Assisted Laser Desorption/Ionization Mass Spectrometry (MALDI-MS), as depicted in **Figure S1** and **Figure S2**.

Synthesis of $PdAu_{24}(2PET)_{18}$

The synthesis of $PdAu_{24}(2PET)_{18}$ was performed as described by Takano et al.[2] Unlike in the original report, cluster isolation was performed with a SiO_2 chromatography column instead of Al_2O_3.

The UV/visible spectrum of the product is presented in **Figure S1**. The synthesized clusters were characterized through MALDI-MS, as depicted in **Figures S2**, **S3**.

Synthesis of Cu/ZnO/Al_2O_3 WGSR benchmark catalyst

The zincian malachite precursor with a molar Cu:Zn:Al ratio of 68:29:3 was synthesized by co-precipitation (T = 338 K) from a Cu, Zn, Al nitrate solution (1.0 M metal-based) and Na_2CO_3 solution (1.6 M) as precipitating agent in an automated lab reactor (OptiMax, Mettler Toledo) at constant pH of 6.5. The precipitate was aged in the mother liquor, filtered, washed, and dried. Calcination was carried out in static air for 3 h, with a heating rate of 2 K min^{-1} and up to 623 K. The catalyst meets the specifications outlined in the FHI standard, ensuring optimal performance and reliability for our intended applications.[3]

MALDI-MS

Matrix-Assisted Laser Desorption Ionization Mass Spectrometry (MALDI-MS) analysis was conducted utilizing a JOEL JMSS3000 spectrometer equipped with a semiconductor laser (λ = 349 nm) and a spiral time-of-flight detector. The matrix employed for the analysis was trans-2-[3-(4-tert-butylphenyl)-2-methyl-2-propenylidene]malononitrile (DCTB). As synthesized $Au_{25}(2PET)_{18}$ clusters were measured in ion positive mode, while any other spectrum was measured in ion negative mode.

UV/Vis absorption spectroscopy

UV/Vis absorption spectra were taken on a JASCO V-670 spectrometer in solution (toluene, THF or DCM).

Cluster immobilization on the ZnO support

Loading

Inductively coupled plasma mass spectrometry (ICP-MS) was used to determine the Au concentration in a cluster–acetone solution. Specifically, 100 µL of the solution was transferred to a glass vial, evaporating the solvent. The resulting clusters were then dissolved in 500 µL of concentrated aqua regia, transferred into a 10 mL volumetric flask, and filled with milliQ water. The solution was subjected to ICP-MS analysis and compared to a standard series.

Based on the concentration, the corresponding volume of the cluster solution was added to the calculated amount of ZnO in suspension. Following 1 h of stirring, the loaded support was separated by centrifugation and washed with a defined volume of acetone. Three distinct samples were prepared, representing different weight ratios (0.1 wt.%, 0.5 wt.%, and 1.0 wt.%). Additionally, the remaining concentrations of Au in the liquid phase were measured by ICP-MS. Detailed results are presented in **Table S1**.

Table S1. Quantitative analysis of immobilization of targeted 0.1 wt.%, 0.5 wt.%, and 1.0 wt.% of $Au_{25}(2PET)_{18-y}(pMBA)_y$ on ZnO.

Sample	Degree of immobilization / %
0.1 wt.%	97.28
0.5 wt.%	97.94
1.0 wt.%	81.54

Ligand exchange

To increase the interaction between the clusters and ZnO during immobilization, 2PET ligands were partially exchanged by p-Mercaptobenzoic acid (pMBA). The thiol end group of pMBA binds to the cluster, while the carboxylic group can bind to ZnO. Aliquots of 3 mg cluster were dissolved in 1 ml acetone, and 3 mg of pMBA ligand was added. After sonication for approximately 5s, the reaction mixture was left at room temperature without stirring for 2h. Acetone was removed by rotary evaporation, and the precipitant was washed five times with a water-methanol mix (3:7). The suspension was centrifuged, and the aqueous phase was removed by decantation, followed by cluster extraction using acetone. The procedure was repeated twice, and MALDI-MS was used to monitor the degree of ligand exchange.

pMBA reacts rapidly with ZnO upon contact, so cluster loss is not a concern during the immobilization step itself. However, a key challenge is obtaining the correct cluster concentration after Cu doping, as the doping process can reduce cluster stability and lead to losses during subsequent handling

(S)TEM

TEM

Transmission electron microscopy (TEM) images were recorded with a JEM-2100 electron microscope (JEOL) operating at 200 kV (**Figures S5**, **S8**).

STEM-HAADF

High-angle annular dark-field scanning transmission electron microscopy (HAADF-STEM) images (**Figure 3**, and **Figures S9**, **S13-S15**) were acquired using a 200 kV FEI Tecnai F20 S-TWIN analytical (scanning) transmission electron microscope [(S)TEM], which was equipped with a Gatan GIF Tridiem filter. The instrument specifications included an energy resolution of ≤1 eV, a semiconvergence angle of ~8 mrad, a semicollection angle of ~15 mrad, and a spatial resolution of 0.5 nm. Catalysts were deposited on carbon-coated copper grids for imaging.

XPS

X-ray photoelectron spectroscopy (XPS) measurements were carried out within a UHV system (base pressure: 5.0×10^{-10} mbar) equipped with a Phoibos 100 hemispherical analyzer and XR 50 X-ray source (SPECS GmbH). The catalyst powder samples were placed on transferable Mo sample holders using UHV-compatible conductive carbon tape. XP spectra were measured in normal emission at room temperature applying Al K_{α} radiation (1486.61 eV), a step size of 0.1 eV, a dwell time of 0.5 s, and a pass energy of 20 eV with the energy analyzer operating in "large area" transmission mode.

For data evaluation, the CasaXPS software package (ver. 3.0) was employed. All spectra were referenced to the C 1s signal (C-C, 284.6 eV), fitted utilizing asymmetric Lorentz as well as Gauss-Lorentz sum functions, and Shirley backgrounds were subtracted. Doublet separations of Au 4f (3.7 eV), Au 4d (18.1 eV), Cu 2p (19.8 eV), Pd 3d (5.2 eV), and Zn 3p (3.0 eV) regions were applied according to the NIST XPS database, and the expected peak area ratios were constrained (Au $4f_{7/2}$:$4f_{5/2}$ = 4:3, Au $4d_{5/2}$:$4d_{3/2}$ = 3:2, Cu $2p_{3/2}$:$2p_{1/2}$ = 2:1, Pd $3d_{5/2}$:$3d_{3/2}$ = 3:2, and Zn $3p_{3/2}$:$3p_{1/2}$ = 2:1).

Table S2. Chemical composition of SAAs on ZnO support, as-prepared and after WGS.

sample	Au / at%*		Pd / at%*		Cu / at%*		S / at%*	
	as-prep.	after WGS	as-prep.	after WGS	as-prep.	after WGS	as-prep.	after WGS
Au	7.7	4.3	-	-	-	-	9.0	3.6
PdAu	5.7	4.3	0.20	0.11	-	-	5.4	4.0
CuAu	3.7	2.4	-	-	0.34	0.28	6.7	2.6
CuPdAu	1.7	1.1	0.09	0.06	0.23	0.17	2.7	1.8

* referenced to the ZnO support

Table S3. Binding energy shifts of SAAs supported on ZnO after pre-treatment compared to the as-prepared state.

sample	Binding energy shift / eV			
	Au 4d	Au 4f	Cu 2p	Pd 3d
Au	-0.4 (Au^0)	-0.4 (Au^0)	-	-
PdAu	-0.3 (Au^0)	-0.3 (Au^0)	-	-0.6 (Pd^0)
CuAu	-	-0.5 (Au^0)	-0.1 (Cu^0/Cu^{x+})	-
CuPdAu	-0.3 (Au^0)	-0.4 (Au^0)	-0.6 (Cu^0/Cu^{x+})	-0.5 (Pd^0)

Table S4. Binding energy shifts of SAAs supported on ZnO after WGSR compared to the pre-treated state.

sample	Binding energy shift / eV			
	Au 4d	Au 4f	Cu 2p	Pd 3d
Au	-0.2 (Au^0)	-0.2 (Au^0)	-	-
PdAu	-0.3 (Au^0)	-0.3 (Au^0)	-	-0.6 (Pd^0)
CuAu	-	-	-0.9 (Cu^0/Cu^{x+})	-
CuPdAu	-	-	-	-0.1 (Pd^0)

Diffuse Reflectance Infrared Fourier Transform Spectroscopy (DRIFTS)

DRIFTS studies were conducted using a Bruker Vertex 70 spectrometer equipped with a liquid nitrogen-cooled MCT (Mercury Cadmium Telluride) detector, offering a spectral resolution of 4 cm^{-1}. An average of 256 scans were taken during the measurements to ensure a robust signal-to-noise ratio. The experimental setup involved a stainless-steel flow cell (Pike) equipped with a CaF_2 window, a cooling system, and an oven. The cell inlet was connected to a gas manifold system featuring calibrated mass flow controllers to precisely adjust the gas mixtures used in the experiments.

The CO adsorption experiments were carried out at 35°C to ensure sample comparability. Initially, the specimens were kept under a continuous He flow at a 47.5 mL/min rate. Infrared spectra were acquired at 3-minute intervals. After collecting at least two spectra in the inert gas environment, CO was introduced at a flow rate of 2.5 mL/min until equilibrium was achieved and the spectra reached a consistent profile. Subsequently, the CO valve was closed, and the gas mixture gradually transitioned back to pure He. The gas content was followed by mass spectrometry.

Pre-treatment conditions

50mg per sample was put under a total flow of 20ml/min of 21vol.% O_2 in inert gas. Once equilibrium was reached and controlled with a mass spectrometer, the temperature was increased with a 10°C/min ramp to the maximum temperature of 300°C. The sample was kept at 300°C for 30 min, followed by a cooldown to 35°C. During cooling, the gases were switched to pure inert gas. Once the flow of 20ml/min of 5 vol.% H_2 reached the equilibrium state, the same temperature program was used for the oxidative pre-treatment.

Total Reflection X-ray Fluorescence (TXRF)

The estimation of cluster catalyst loading on ZnO was carried out utilizing TXRF. This analysis employed an ATOMIKA 8030C X-ray fluorescence analyzer, featuring a total reflection geometry and equipped with an energy-dispersive Si(Li)-detector, boosting an energy resolution of 160 eV. The measurements were executed utilizing a monochromatized Mo K_α excitation mode (17.48 keV). The angle of incidence was set to approximately 70% of the critical angle for total reflection of X-rays, equivalent to about 1.2 mrad. Experimental conditions included a live time of 100 seconds, a voltage of 50 kV, and a current of 47 mA. Notably, elemental proportions were calculated in reference to 100% Zn, implying that the measurements were standardized to the concentration of zinc within the sample. The final loadings of the catalysts were then calculated to the metal oxide.

Table S5. TXRF relative quantification results, calculated individual atom loadings, and factors used for activity normalization.

sample	metal loading / wt.%	Au loading / wt.%	Pd loading / wt.%	Cu loading / wt.%	factor to 1 wt.%
Au	0.36	0.36	-	-	2.77
CuAu	0.20	0.194	-	0.006	4.98
PdAu	0.24	0.235	0.005	-	4.15
CuPdAu	0.08	0.075	0.002	0.003	12.45
Cu-ref.	0.48	0.48	-	-	2.07

Activity Tests

After the oxidative and reductive pre-treatments described in "Pre-treatment conditions" above, the gas mix was changed to the reaction mix at 25°C (0.75ml/min CO and 0.25ml/min H2O, 19ml/min He (total)). H_2O was added by flowing 9.25ml/min He through a bubbler kept at 25°C with a cryostat. The temperature was increased by a ramp of 10°C/min between 25°C and 250°C and then by 5°C/min up to 400°C. At 50°C intervals (50°C, 100°C, 150°C, ..., 400°C), the temperature was kept constant for 5 min to ensure a gas chromatography (GC) datapoint for comparison. In the cool down to room temperature between the two consecutive runs, the samples were kept under reaction gas. After the second run, the samples were cooled under inert gas.

Gas analysis was performed with a MicroGC Fusion 3000A setup from Inficon (Bad Ragatz, Switzerland). For the separation of H_2, O_2, N_2, CH_4, and CO, a Molsieve column with 5 Å pore diameter was used. CO_2 was detected on an RT-Q bond column, and both columns detected the gas with a TCD detector. The temperature programs were set to ensure data at 50°C steps.

Figures

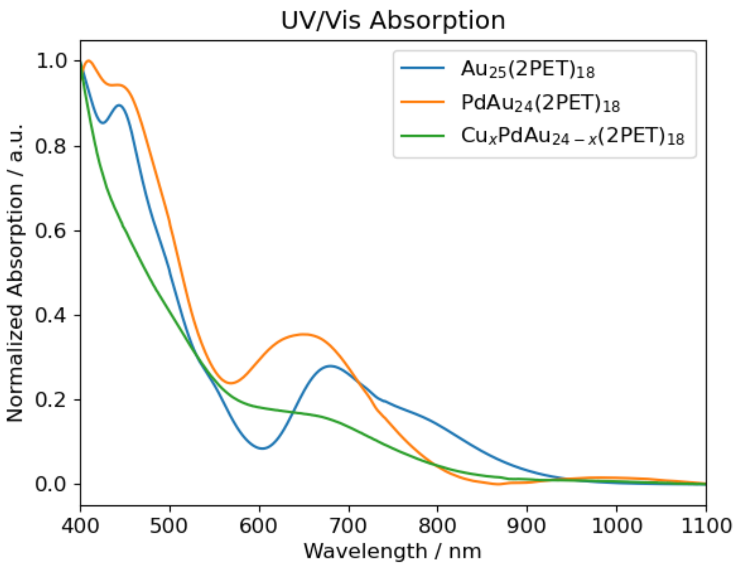

Figure S1. UV/Vis absorption spectra of $Au_{25}(2PET)_{18}$, $PdAu_{24}(2PET)_{18}$, and $Cu_xPdAu_{24-x}(2PET)_{18}$ in the "as prepared" state.

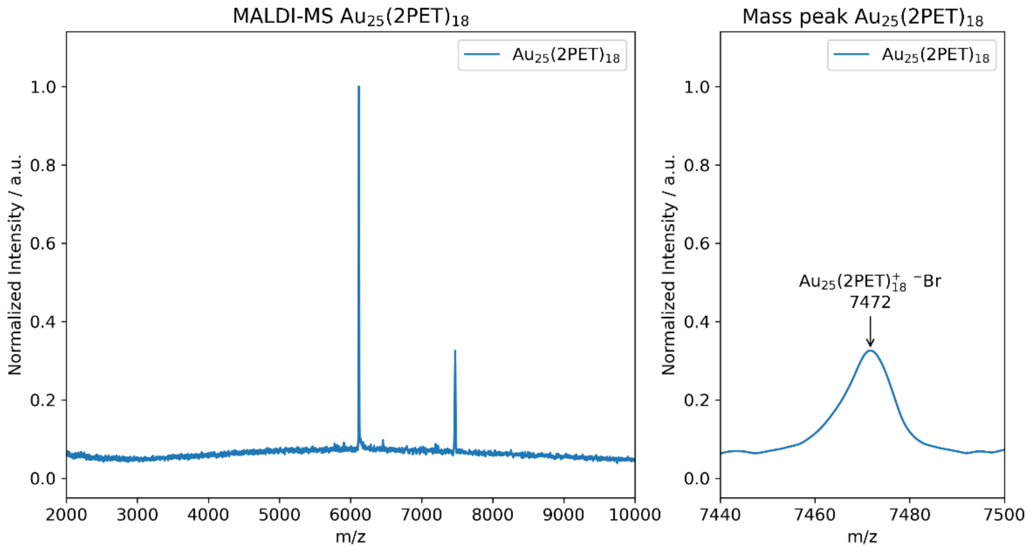

Figure S2. MALDI-MS of $Au_{25}(2PET)_{18}$ to confirm the product purity of the synthesis.

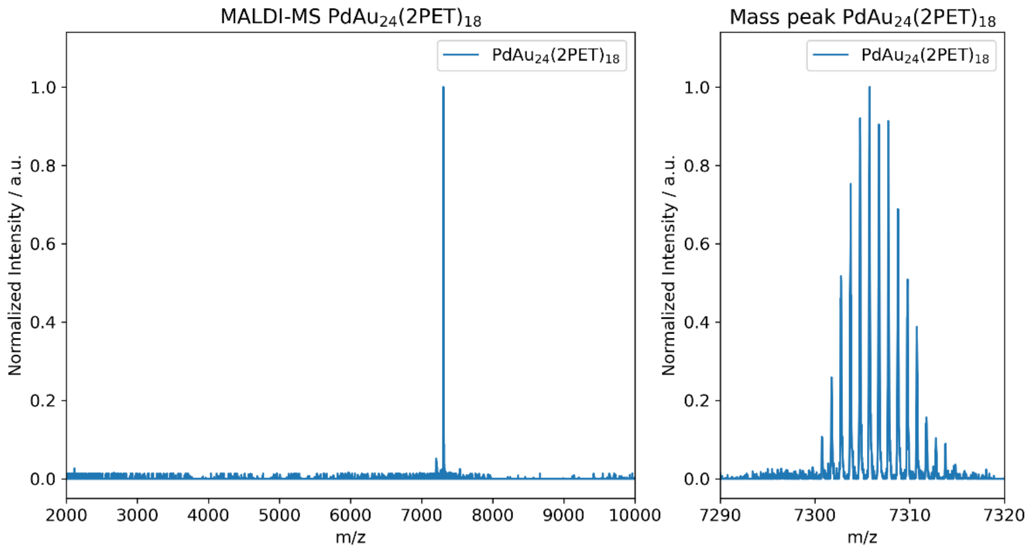

Figure S3. MALDI-MS of $PdAu_{24}(2PET)_{18}$ to confirm the product purity of the synthesis.

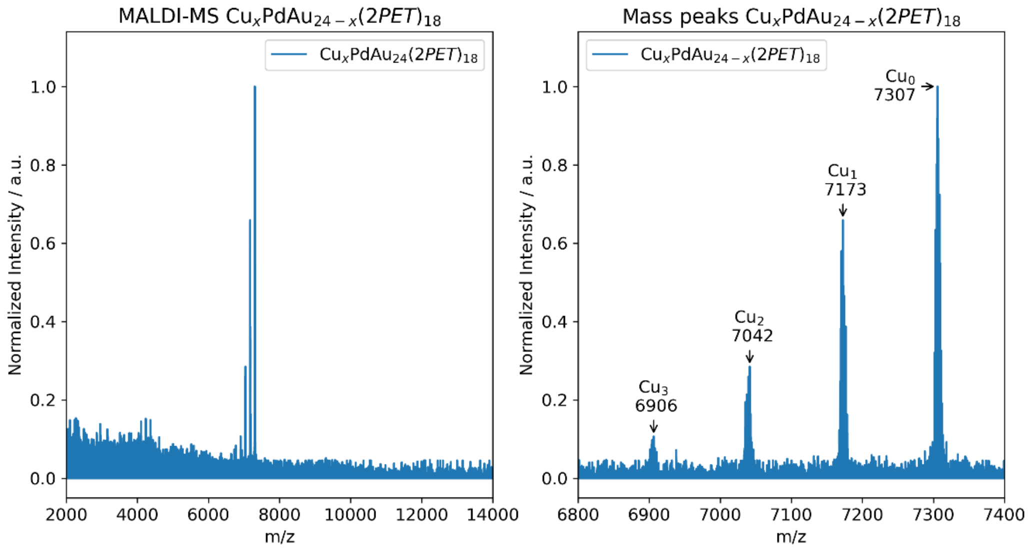

Figure S4. MALDI-MS of $Cu_xPdAu_{24-x}(2PET)_{18}$ (x = 0-3) to confirm the product purity of the synthesis.

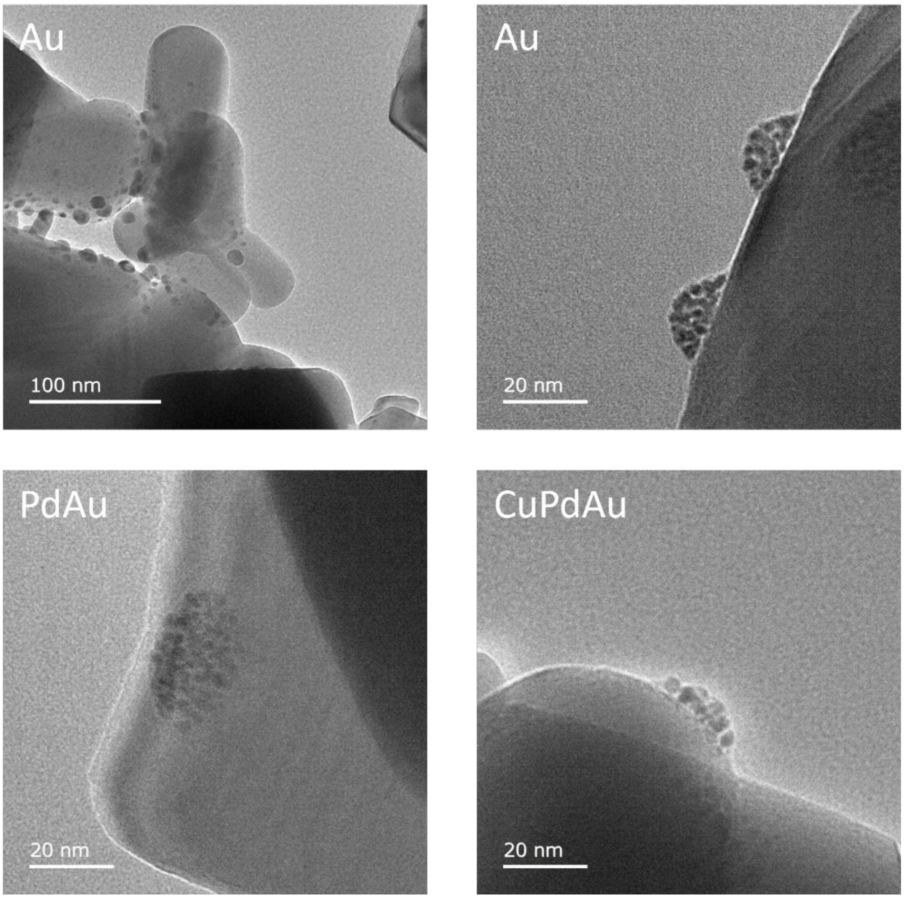

Figure S5. TEM images of agglomeration due to the 2PET ligand of nanoclusters on ZnO. Top: $Au_{25}(2PET)_{18}$, bottom left: $PdAu_{24}(2PET)_{18}$, bottom right: $Cu_xPdAu_{24-x}(2PET)_{18}$.

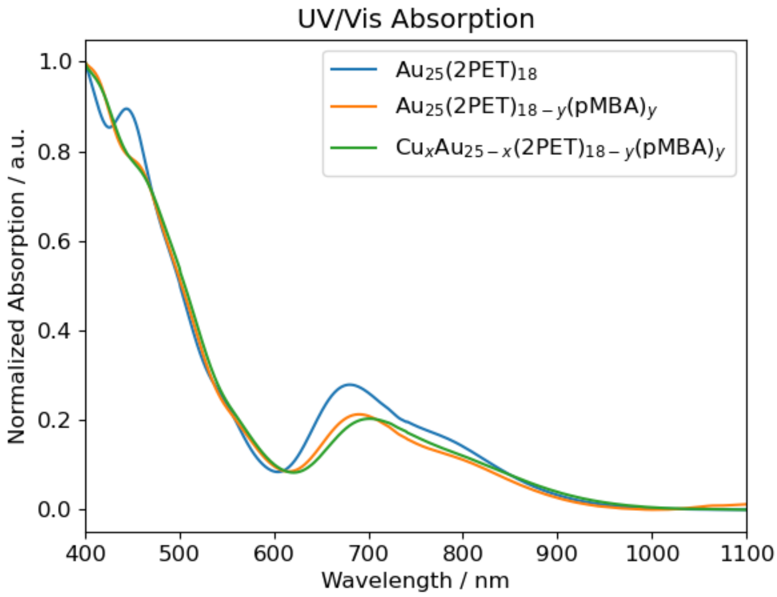

Figure S6. UV/Vis absorption spectra of $Au_{25}(2PET)_{18}$ (blue), after ligand exchange (orange) and after doping yielding $Cu_xAu_{25-x}(2PET)_{18-y}(pMBA)_y$ (green).

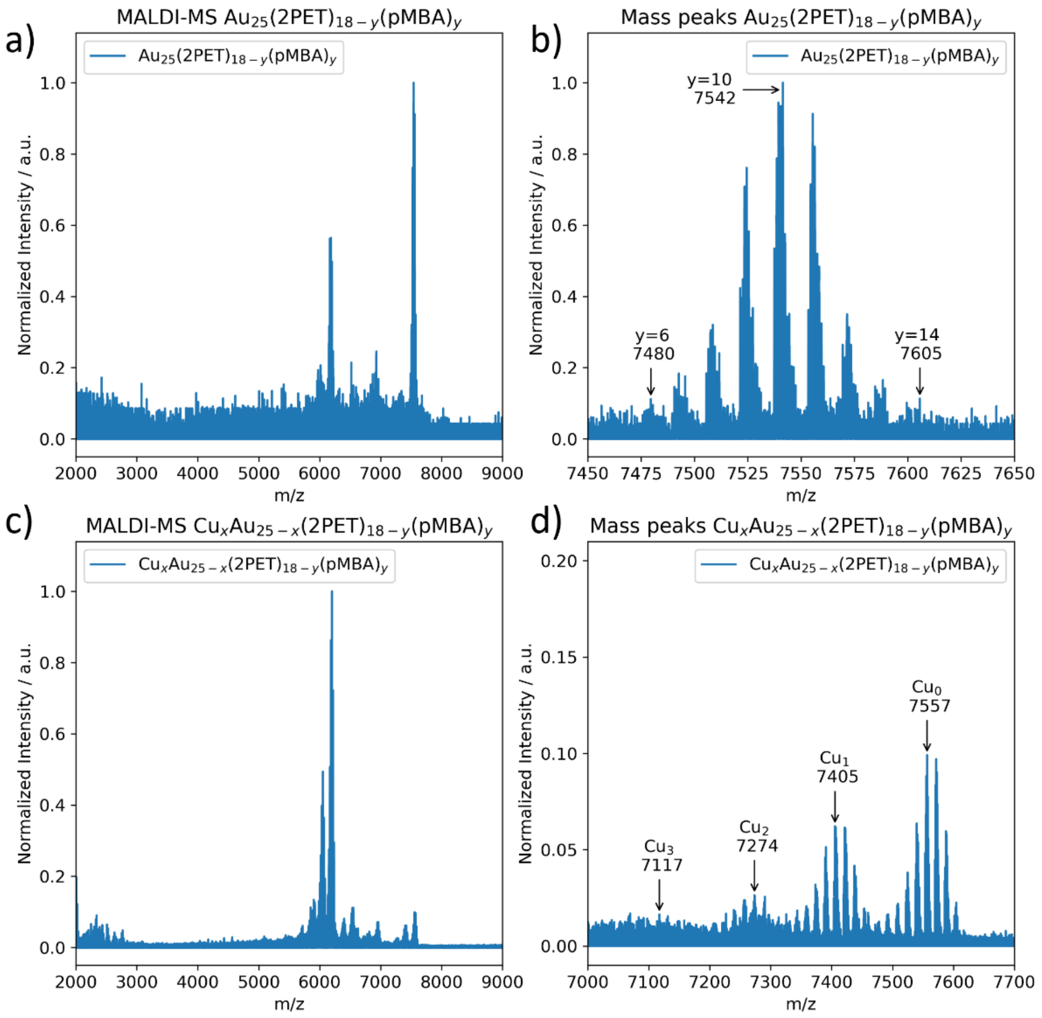

Figure S7. a) MALDI-MS spectrum of $Au_{25}(2PET)_{18-y}(pMBA)_y$ (y=6-14) after ligand exchanged applied to $Au_{25}(2PET)_{18}$, b) mass peaks range of $Au_{25}(2PET)_{18-y}(pMBA)_y$ (y=6-14). Bottom: Cu doped into the $Au_{25}(2PET)_{18-y}(pMBA)_y$ clusters after ligand exchange. c) MALDI-MS spectrum after Cu doping of $Au_{25}(2PET)_{18-y}(pMBA)_y$ to $Cu_xAu_{24-x}(2PET)_{18-y}(pMBA)_y$ (x=0-3, y=5-13). d) mass peak range of $Cu_xAu_{24-x}(2PET)_{18-y}(pMBA)_y$ (x=0-3, y=5-13).

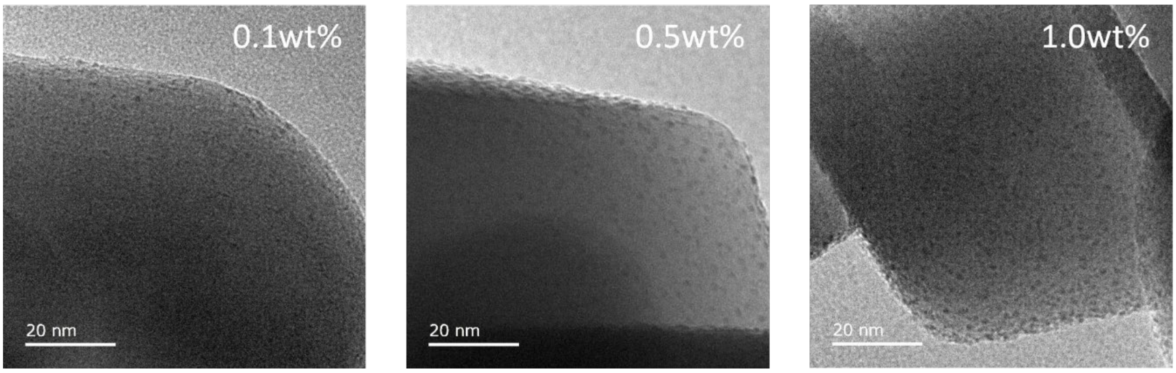

Figure S8. TEM bright field images of the loading optimization of $Au_{25}(2PET)_{18-y}(pMBA)_y$ on ZnO. The supported particles were approximately 1 nm in size for 0.1 and 0.5 wt.% loading and about 2 nm for 1.0 wt.%.

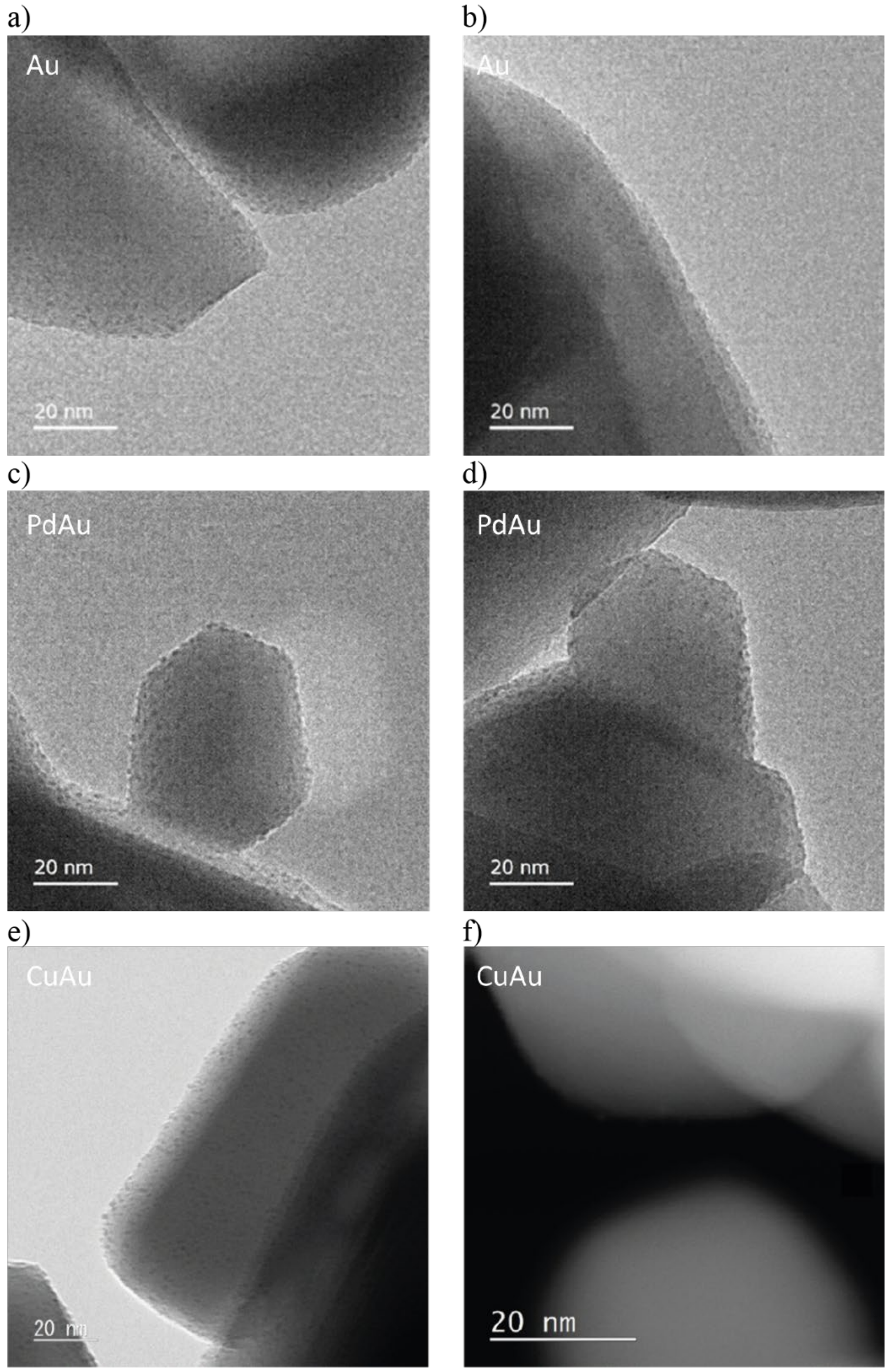

Figure S9. Images of the immobilized clusters on ZnO. a), b) $Au_{25}(2PET)_{18-y}(pMBA)_y$ TEM brightfield, c), d) $PdAu_{24}(2PET)_{18-y}(pMBA)_y$ TEM bright field, and e), f) $Cu_xAu_{25-x}(2PET)_{18-y}(pMBA)_y$ clusters on ZnO ((e): TEM brightfield, f): STEM-HAADF).

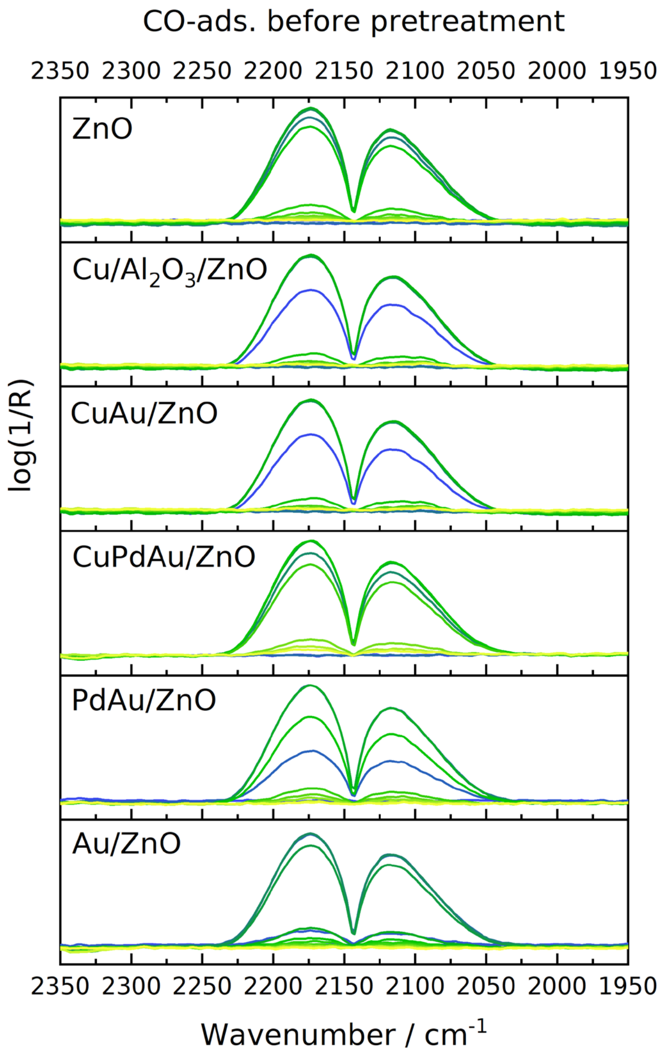

Figure S10. DRIFTS spectra of CO adsorption on the as-prepared catalysts before pre-treatment (blue over green to yellow). Only the CO gas phase double bands can be observed. The references and the cluster catalysts do not show CO absorption prior to pre-treatment. (T=35°C, 1 bar, 47.5ml/min He + 2.5ml/min CO (added and removed)) The stacking of the spectra facilitates to compare the catalysts.

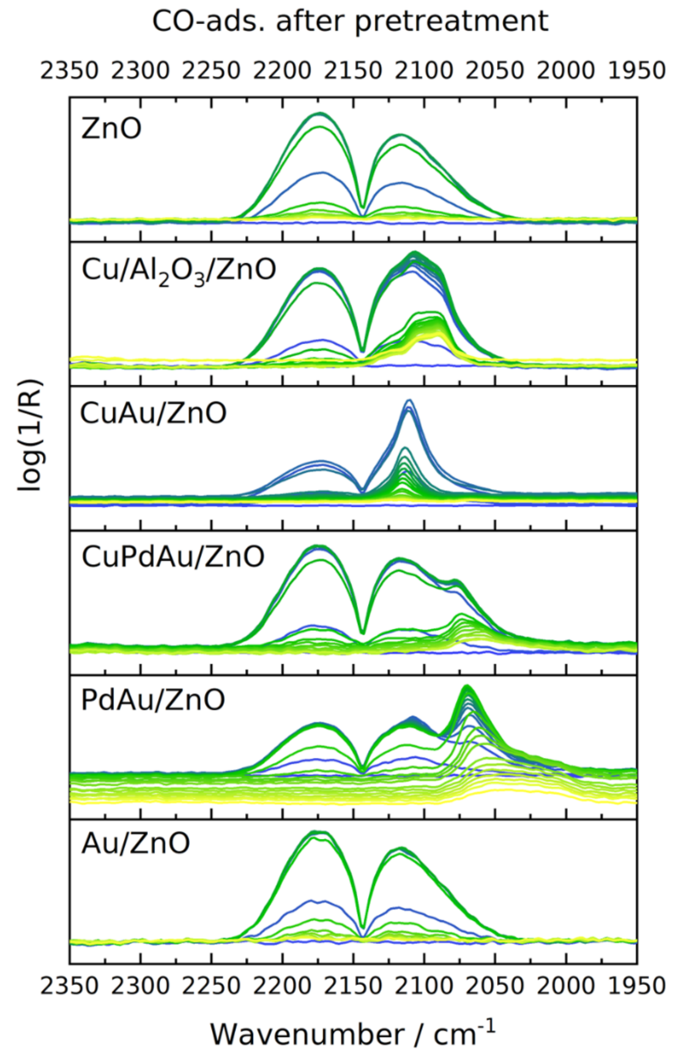

Figure S11. In situ DRIFTS spectra of CO adsorption on pre-treated catalysts (blue over green to yellow). On top of the double band of the CO gas phase, CO adsorption can be observed on the metal dopants and the Cu reference catalyst (T=35°C, 1 bar, 47.5ml/min He + 2.5ml/min CO (added and removed)). The stacking order of the spectra is intended to facilitate catalyst comparison.

CO(g) Background Correction in DRIFTS

For a comprehensive analysis of the infrared (IR) bands, the double peak of gaseous CO was systematically eliminated from the post-pre-treatment spectra. The spectral component located at higher wavenumbers exhibited no overlap with potential adsorption signals. It was used to fit a $CO_{(g)}$ spectrum measured over pure ZnO, taken under the same conditions as the sample catalysts. In the next step, the adjusted CO spectrum was subtracted, leading to difference spectra only containing information from the interaction of CO with the specimens. An example of the procedure is shown in **Figure S12** and the resulting spectra of all samples are shown in **Figure 5**.

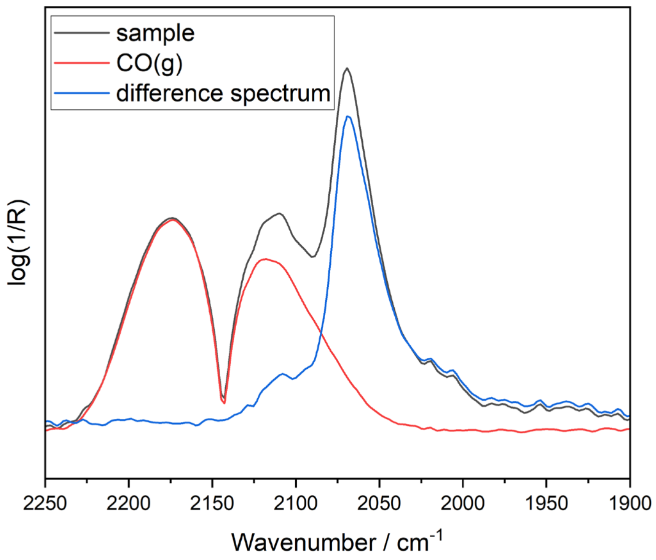

Figure S12. Example of $CO_{(g)}$ spectra subtraction in a CO adsorption experiment.

Gas Hourly Space Velocity (GHSV) and Residence Time (τ)

The GHSV describes the gas flow rate through the catalyst bed in the reactor, equivalent to the inverse of the residence time. The volume of the tube reactor bed was calculated via the catalyst weight (60 mg) and the ZnO density. The metal loading was neglected due to its minimal contribution to the overall density. In addition, a packing of 64% was used for the catalyst bed, assuming random closed packing (RCP):

$$V_{cat.\ bed} = \frac{m_{cat.}}{\rho(ZnO)} \cdot f \quad (1)$$

$$GHSV = \frac{\dot{V}_{total}}{V_{cat.\ bed}} \quad (2)$$

$$\tau = \frac{1}{GHSV} \quad (3)$$

mass of catalyst: $m_{cat.} = 0.050$ g

density of ZnO: $\rho(ZnO) = 5.61 \cdot 10^3$ g · l^{-1}

packing factor: $f = 1.56$

volume of catalyst bed: $V_{cat.\ bed} = 1.4 \cdot 10^{-5}$ l

total flow of gases: $\dot{V}_{total} = 20$ ml · min^{-1} = 1.2 l · h^{-1}

Gas Hourly Space Velocity: $GHSV = 8.6 \cdot 10^4$ h^{-1}

Residence Time: $\tau = 1.2 \cdot 10^{-5}$ h = 0.042 s

Space-Time Conversion (STC)

The STC is a parameter that characterizes catalyst performance based on the conversion of the educt CO. First, the volume of CO in the catalyst bed is calculated during the residence time, representing the maximum number of mol of CO present when conversion is zero, based on the universal gas equation. The STC values are finally obtained by inserting the conversion as measured by calibrated GC. The $\overline{STC}$ is normalized to the metal loading to compare the activity based on the metal/alloy nanoparticles. This is justified as, due to the small cluster size, nearly all metal atoms are located on the particle's surface. For the technological Cu/ZnO/Al_2O_3 catalyst, STEM images confirmed the redistribution of Cu to single atoms or particles below the detection limit, thus all accessible by the educts.

$$V_{CO} = \dot{V}_{CO} \cdot \tau \qquad (4)$$

$$n_{CO} = \frac{p \cdot V_{CO}}{R \cdot T} \qquad (5)$$

$$m_{CO} = n_{CO} \cdot M_{CO} \qquad (6)$$

$$STC = \frac{\text{conversion} \cdot m_{CO}}{100 \cdot m_{cat} \cdot \tau} \qquad (7)$$

$$\overline{STC} = \frac{\text{conversion} \cdot m_{CO}}{100 \cdot m_{cat} \cdot \tau \cdot \text{loading}} \qquad (8)$$

Total flow of CO: $\dot{V}_{CO} = 0.045\ \mathrm{l \cdot h^{-1}}$

Residence Time: $\tau = 1.2 \cdot 10^{-5}\ \mathrm{h} = 0.042\ \mathrm{s}$

Volume CO in the catalyst bed: $V_{CO} = 5.2 \cdot 10^{-7}\ \mathrm{l}$

Pressure: $p = 1\ \mathrm{bar}$

Universal gas constant: $R = 0.08314\ \mathrm{l \cdot bar \cdot mol^{-1} \cdot K^{-1}}$

Temperature: $T = 298.15\ \mathrm{K}$

Maximum numbers of mol CO in the catalyst bed: $n_{CO} = 2.1 \cdot 10^{-8}\ \mathrm{mol}$

$M_{CO} = 28.01\ \mathrm{g \cdot mol^{-1}}$

Maximum mass of CO in the catalyst bed: $m_{CO} = 5.9 \cdot 10^{-7}\mathrm{g}$

Space-time conversion $STC\ in\ \mathrm{g}_{CO} \cdot \mathrm{g}_{cat}{}^{-1} \cdot \mathrm{h}^{-1}$

Metal loading in wt.%

STC normalized to total metal loading $\overline{STC}\ in\ \mathrm{g}_{CO} \cdot \mathrm{g}_{cat}{}^{-1} \cdot \mathrm{h}^{-1} \cdot \mathrm{wt.\%}^{-1}$

Table S6. Results of STC calculations based on CO conversion at 400°C.

catalyst	loading / wt.%	conversion / %		STC / 10^{-2} g_{CO} *g_{cat}^{-1}*h^{-1}		$\overline{STC}$ / 10^{-2}*g_{CO} *g_{cat}^{-1}*h^{-1}* wt.%$^{-1}$	
		1st run	2nd run	1st run	2nd run	1st run	2nd run
Au/ZnO	0.36	3.01	3.25	3.1	3.3	8.5	9.2
CuAu/ZnO	0.20	5.88	4.72	6.0	4.8	30	24
Cu-ref.	0.48	6.45	5.90	6.6	6.0	14	13
CuPdAu/ZnO	0.08	5.16	5.33	5	5	70	70
PdAu/ZnO	0.24	5.27	5.16	5.4	5.3	22	22

Space Time Yield STY

To calculate the space-time yield for hydrogen, equation (7) is modified to equation (10). No side products were detected, so yield equals conversion. Again, the STY was normalized to the catalysts' loading to allow comparison among the SAAs. The maximum mass of hydrogen is calculated based on the maximum mol of CO in case of 100% conversion.

$$m_{H_2} = n_{CO} \cdot M_{H_2} \qquad (9)$$

$$STY = \frac{\text{yield} \cdot m_{H_2}}{100 \cdot m_{cat} \cdot \tau} \qquad (10)$$

$$\overline{STY} = \frac{\text{yield} \cdot m_{H_2}}{100 \cdot m_{cat} \cdot \tau \cdot \text{loading}} \qquad (11)$$

Table S7. Results of STY calculations based on H_2 yield at 400°C.

catalyst	yield H_2 / %		STY / 10^{-3} g_{H2} *g_{cat}^{-1}*h^{-1}		$\overline{STY}$ / 10^{-2}*g_{H2} *g_{cat}^{-1}*h^{-1}* wt.%$^{-1}$	
	1st run	2nd run	1st run	2nd run	1st run	2nd run
Au/ZnO	3.01	3.25	2.2	2.4	0.61	0.66
CuAu/ZnO	5.88	4.72	4.3	3.5	2.2	1.7
Cu-ref.	6.45	5.90	4.7	4.3	0.98	0.90
CuPdAu/ZnO	5.16	5.33	4	4	5	5
PdAu/ZnO	5.27	5.16	3.9	3.8	1.6	1.6

Turnover Frequency TOF

Table S8 additionally compares the turnover frequencies (TOFs; molecules CO per “cluster” per second) for the different catalysts, although this has to be taken with care. Clearly, heteroatom doping drastically increased the activity, but the active site has not yet been identified. Hence, further breakdown (normalization) to specific (single) atoms was not undertaken for TOF calculations. The clusters’ ligands have been removed during pre-treatment and are thus irrelevant for the calculation. As the number of atoms per cluster is exactly known, the number of clusters can be calculated based on the metal loading determined by TXRF and the total mass of the used catalyst. With the total flow of CO, the flow of mol CO is first calculated, followed by the flow of CO molecules. This flow calculates the turnover frequency with the CO conversion normalized per cluster per second. The first reaction run was used to calculate the TOF per cluster. TOF values were calculated at 400°C under differential conditions with CO conversions below 20%, as reported in **Table S8**. TOFs were normalized per cluster (25 atoms) rather than per heteroatom dopant to avoid exaggerated values and ensure consistent comparison across catalysts.

$$m_{loading} = \frac{\text{loading} \cdot m_{cat.}}{100} \text{g} \tag{12}$$

$$m_{element} = \frac{M_{element}}{N_A} \text{g} \tag{13}$$

$$m_{cluster} = (25 - x - y) \cdot m_{Au1} + x \cdot m_{Pd1} + y \cdot m_{Cu1} \text{ g} \tag{14}$$

$$N_{cluster} = \frac{m_{loading}}{m_{cluster}} \tag{15}$$

$$TOF = \frac{\text{conversion} \cdot \dot{z}_{CO}}{100 \cdot N_{cluster}} s^{-1} \tag{16}$$

mass of catalyst: $m_{cat.} = 0.050$ g

mass of metal loading $m_{loading}$ in g

mass of one atom $m_{element}$ in g (element: Au, Pd, Cu)

mass metal per cluster in g (x, y: **Table S8**)

number of “clusters” $N_{cluster}$

Total flow of CO: $\dot{V}_{CO} = 1.25 \cdot 10^{-5}\ \text{l} \cdot \text{s}^{-1}$

Volume mol: $V_{mol} = 22.414\ \text{l} \cdot \text{mol}^{-1}$

Molar flow CO: $\dot{n}_{CO} = \frac{\dot{V}_{CO}}{V_{mol}} = 5.6 \cdot 10^{-7}\ \text{mol} \cdot \text{s}^{-1}$

Flow CO molecules: $\dot{z}_{CO} = \dot{n}_{CO} \cdot N_A = 3.4 \cdot 10^{17}\ \text{s}^{-1}$

Turnover frequency TOF in converted molecules CO per cluster s^{-1}

Table S8. TOF (CO molecules converted per "cluster" per second) of cluster-based SAA catalysts and values for the cluster mass calculations in Equation 14.

Sample	x	y	TOF 1st run / s^{-1}
Au/ZnO	0	0	0.46
CuAu/ZnO	0	3	1.5
CuPdAu/ZnO	1	3	3
PdAu/ZnO	1	0	1.2

(S)TEM Particle size distribution

Table S9. The cluster size distribution of the SAAs in the as-prepared state, before and after WGSR.

Sample	As-prepared [nm]	σ [nm]	Before WGSR [nm]	σ [nm]	After WGSR [nm]	σ [nm]
**Au**	**1.0**	**±0.3**	**1.6**	**±0.7**	**2.2**	**±0.8**
**PdAu**	**1.0**	**±0.2**	**1.5**	**±0.4**	**2.2**	**±0.9**
**CuAu**	**1.1**	**±0.3**	**1.4**	**±0.4**	**1.8**	**±1.0**
**CuPdAu**	**0.9**	**±0.2**	**1.9**	**±0.7**	**1.7**	**±0.8**

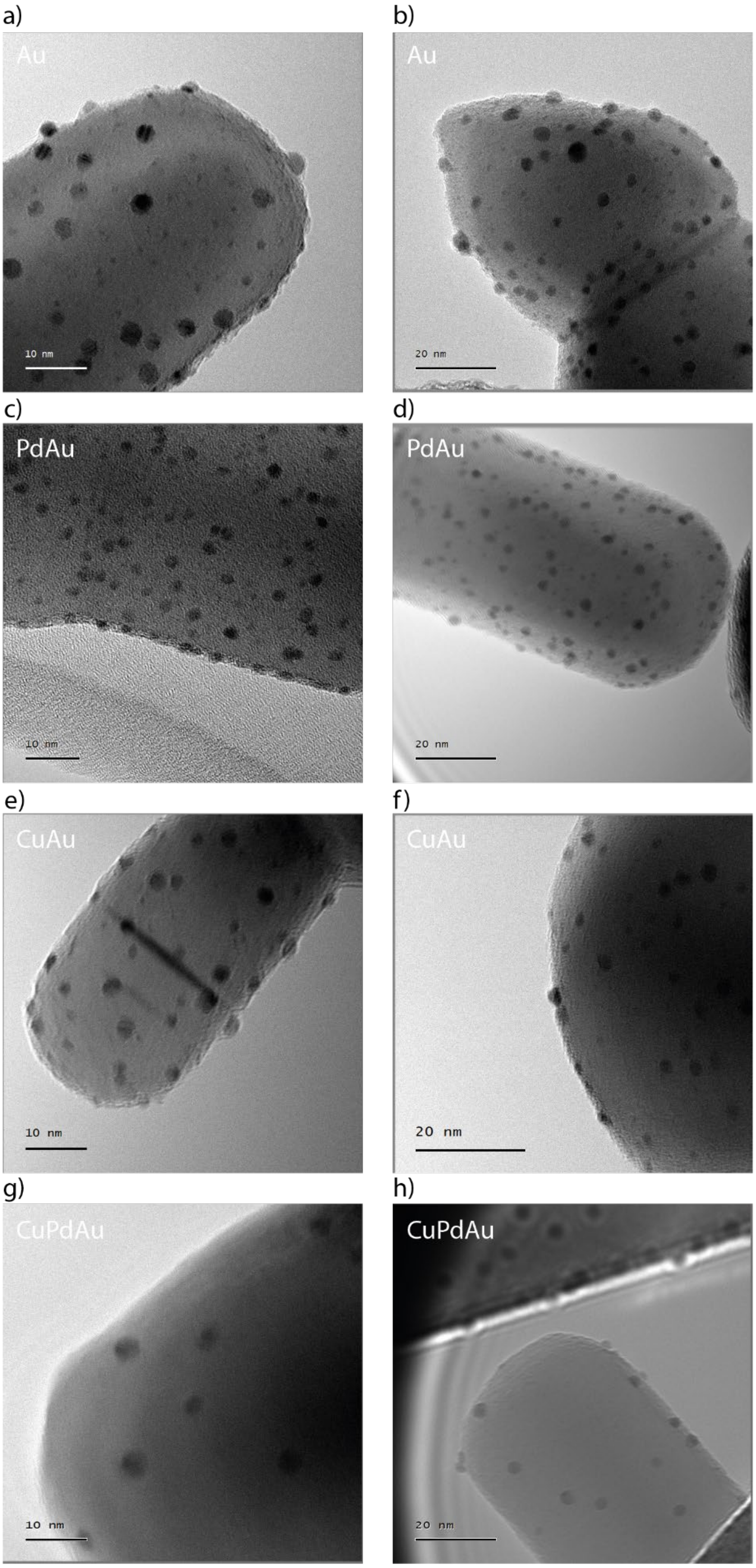

Figure S13. TEM brightfield images of the SAA catalyst after the pre-treatment and before WGS reaction.

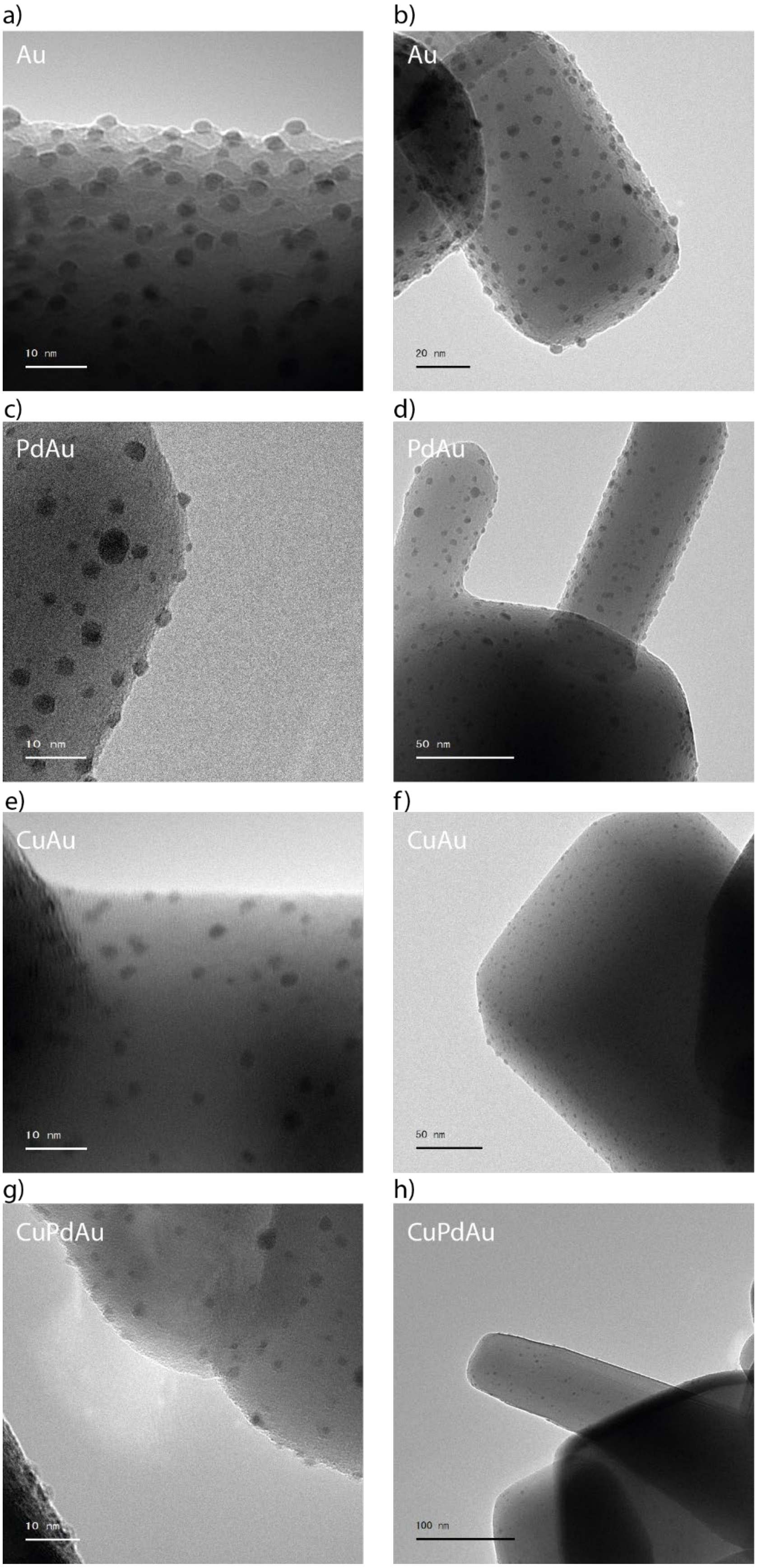

Figure S14. TEM brightfield images of the SAA catalyst after the WGS reaction.

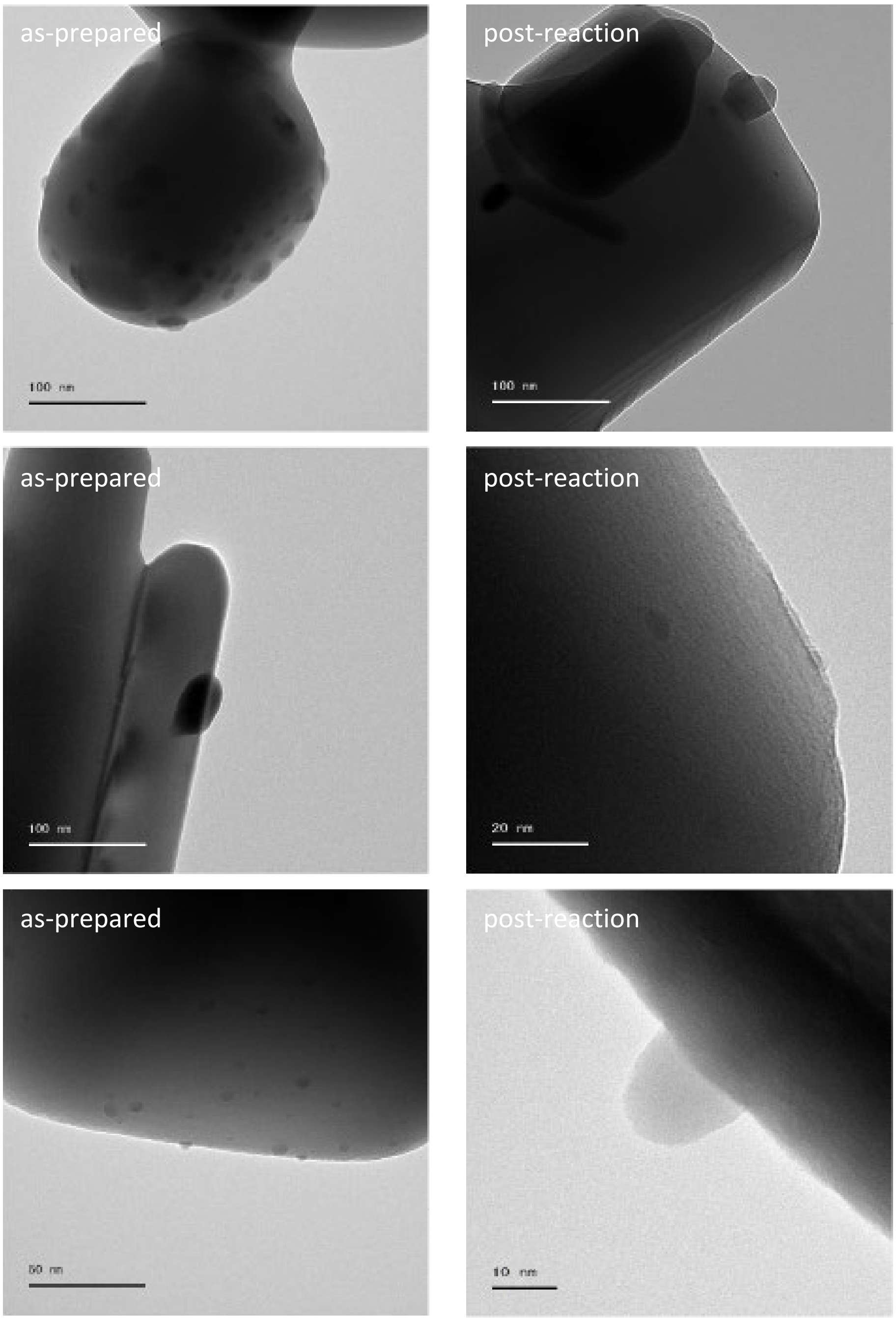

Figure S15. TEM bright field images of the industrial $Cu/Al_2O_3/ZnO$ reference catalyst. Before reaction (left column), after reaction (right column).

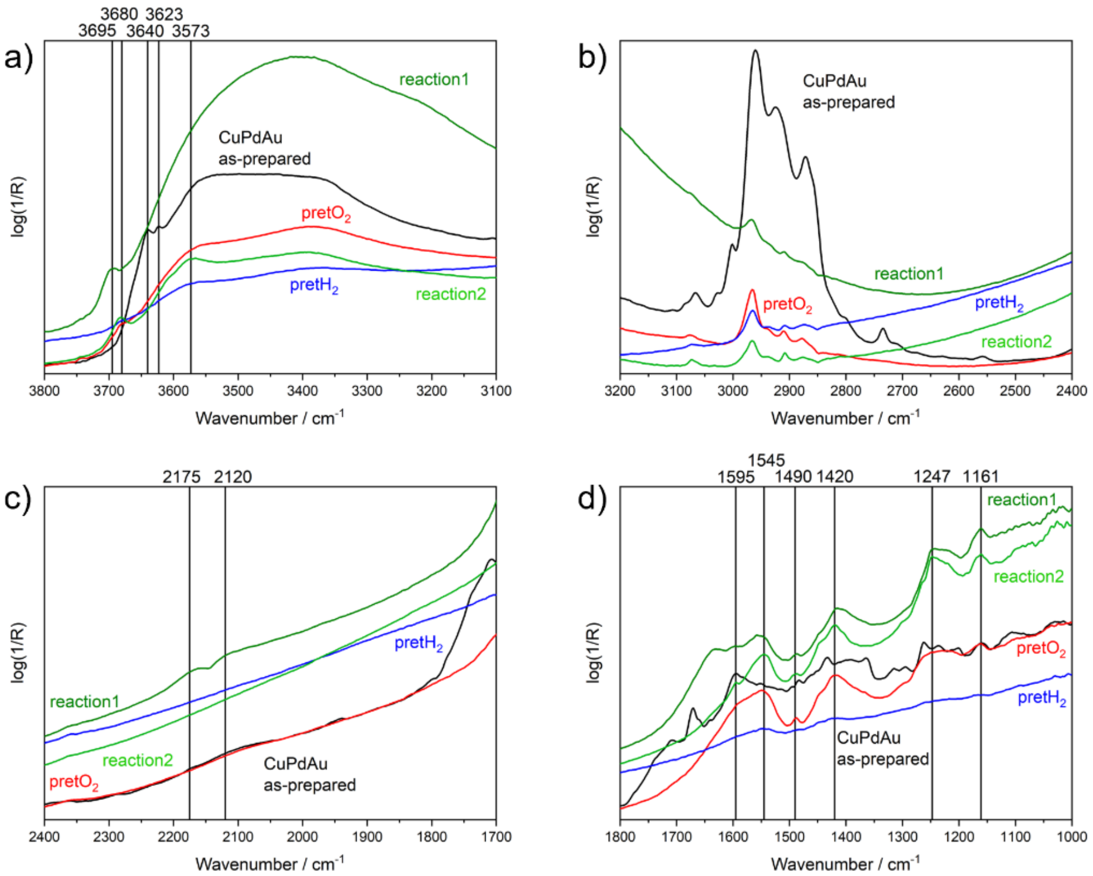

Figure S16. *In situ* DRIFTS measurements of the CuPdAu SAA as-prepared (black, He), after O_2 pre-treatment (red, He), after H_2 pre-treatment (blue, He), after first reaction run (dark green, reaction gas mix), and after the second reaction run (light green, He). For readability, the spectra are split into the regions a) 3800-3100 cm^{-1}, b) 3200-2400, c) 2400-1700 cm^{-1}, and d) 1800-100 cm^{-1}.

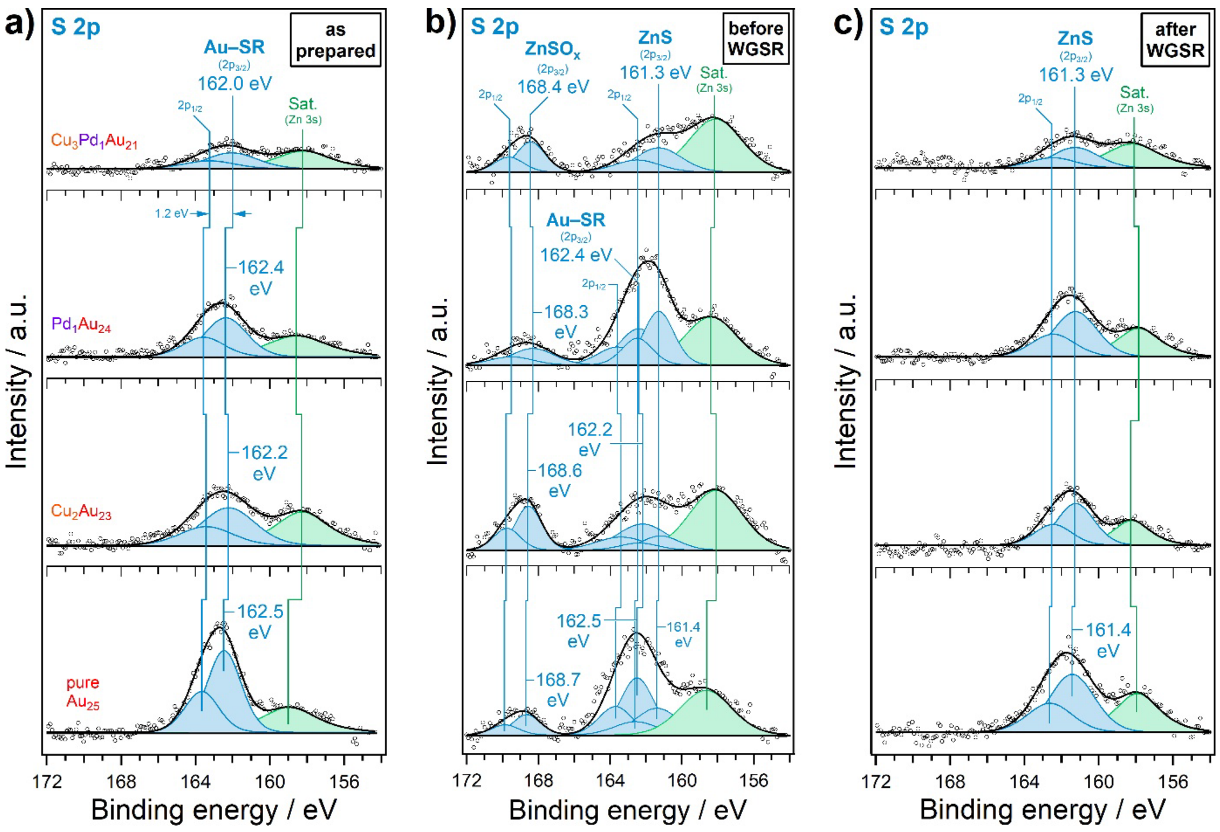

Figure S17. XPS spectra of the S 2p region of the SAA catalysts. a) In the as-prepared state, b) after pre-treatment, and c) after WGS reaction.

References

(1) Brust, M.; Walker, M.; Bethell, D.; Schiffrin, D. J.; Whyman, R. Synthesis of thiol-derivatised gold nanoparticles in a two-phase Liquid–Liquid system. *Journal of the Chemical Society, Chemical Communications* **1994**, (7), 801-802. DOI: 10.1039/C39940000801.

(2) Takano, S.; Ito, S.; Tsukuda, T. Efficient and Selective Conversion of Phosphine-Protected $(MAu_8)^{2+}$ (M = Pd, Pt) Superatoms to Thiolate-Protected $(MAu_{12})^{6+}$ or Alkynyl-Protected $(MAu_{12})^{4+}$ Superatoms via Hydride Doping. *Journal of the American Chemical Society* **2019**, *141* (40), 15994-16002. DOI: 10.1021/jacs.9b08055.

(3) Heenemann, M.; Millet, M.-M.; Girgsdies, F.; Eichelbaum, M.; Risse, T.; Schlögl, R.; Jones, T.; Frei, E. The Mechanism of Interfacial CO_2 Activation on Al Doped Cu/ZnO. *ACS Catalysis* **2020**, *10* (10), 5672-5680. DOI: 10.1021/acscatal.0c00574.
